# A Peripheral Mechanism of Depression: Disturbed Intestinal Epithelial *Per2* Gene Expression Causes Depressive Behaviors in Mice with Circadian Rhythm Disruption via Gut Barrier Damage and Microbiota Dysbiosis

**DOI:** 10.1002/advs.202501818

**Published:** 2025-08-23

**Authors:** Huiliang Zhang, Xuan Qin, Haiyue Song, Jieru Zhou, Hui Wei, Lun Zhang, Yi Liu, Zhuoqun Wang, Yiren Zhang, Yiwen Lai, Jiayu Yang, Wenting Hu, Zhongshan Chen, Ji Zeng, Yu Jin, Xiaochuan Wang, Rong Liu

**Affiliations:** ^1^ Department of Pathophysiology Key Laboratory of Ministry of Education/Hubei province for Neurological Disorders School of Basic Medicine Tongji Medical College Huazhong University of Science and Technology Wuhan 430030 China; ^2^ Division of Gastroenterology Union Hospital Tongji Medical College Huazhong University of Science and Technology Wuhan 430022 China; ^3^ Taikang Tongji Hospital Wuhan 430050 China; ^4^ Department of Clinical Laboratory Wuhan Fourth Hospital Wuhan 430033 China; ^5^ Department of Pathology Peking University Shenzhen Hospital Shenzhen 518036 China; ^6^ Shenzhen Huazhong University of Science and Technology Research Institute Shenzhen 518038 China; ^7^ Hubei Key Laboratory of Cognitive and Affective Disorders Institute of Biomedical Sciences School of Medicine Jianghan University Wuhan 430200 China; ^8^ Institute for Brain Research Wuhan Center of Brain Science Huazhong University of Science and Technology Wuhan 430030 China

**Keywords:** circadian rhythm disruption, depression, gut barrier, gut microbiota, neurogenesis, neuroinflammation, *Per2*

## Abstract

Circadian rhythm disruption (CRD) is a potential risk factor for the development of depression. However, the underlying mechanisms remain unclarified. Here, it is found that in CRD model mice showing significant depressive‐like behaviors, the expression rhythm of *Period 2* (*Per2*), an important rhythm gene, is disrupted in intestinal epithelium, which results in defect of gut barrier integrity and gut microbiota disturbance, accompanied by peripheral and neuroinflammation, deficit in hippocampal neurogenesis, and impairment of excitatory neurotransmission. Specific knockdown of *Per2* gene in intestinal epithelial cells prevents the development of depression‐like phenotype induced by CRD, with a reverse of these pathologic changes. Metabonomic analysis reveals that both CRD and CRD gut microbiota‐transplanted mice have downregulated tryptophan metabolism and reduced tryptophan levels both in serum and brain, and tryptophan supplementation is sufficient to prevent CRD‐induced depression, reduce systemic and neuronal inflammatory response, and rescue neurogenesis and synaptic function. These data suggest that the disturbed expression of intestinal epithelial *Per2* gene plays a critical role in CRD‐induced neurological damage and depression in mice, which is mediated by gut microbiota and metabolites. Therefore, specific targeting on intestinal epithelial *Per2* or tryptophan metabolism is a promising strategy to prevent CRD‐induced depression.

## Graphical abstract

Circardian rhythm disorder (CRD) causes abnormal expression of intestinal Period 2 (Per2) gene, which compromising intestinal barrier integrity and altering the gut microenvironment. Microbiota dysbiosis and aberrant metabolites production drive central inflammation, impair neurogenesis, and promote functional deficits, ultimately facilitating the development of depression.

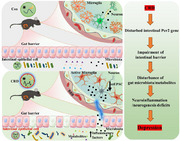

## 1 Introduction

Major depressive disorder (MDD) is a prevalent mental illness characterized by various physical changes, including depressed mood, anhedonia, drowsiness, difficulty in concentrating, and appetite loss. It is also a leading cause of suicide. According to the World Health Organization, MDD is projected to become the leading cause of disability worldwide by 2030.^[^ ^1^ ^]^ Currently, the etiology and pathogenesis of MDD remain unclear, and available clinical treatments and interventions are limited. Exploring the pathogenesis of depression and identifying effective targets for pharmacological interventions are urgent and critical social and medical concerns.

Most physiological processes in the human body exhibit circadian rhythms, which coordinate essential functions such as the sleep–wake cycle, locomotor activity, body temperature, hormone secretion, and energy metabolism. In modern life, circadian rhythm disruption (CRD) has become increasingly common, primarily due to factors such as job rotation, jet lag, untimely light exposure, and advancing age. Numerous population‐based epidemiologic and animal studies have demonstrated a strong association between CRD and the development of depression. A meta‐analysis comprising 11 studies concluded that night‐shift workers are 40% more likely to experience depression compared to their daytime counterparts.^[^ ^2^ ^]^ Furthermore, results from several large population‐based surveys including studies involving a cohort of 11 450 nurses, 14 000 night‐shift workers, and 4000 flight attendants, have revealed a robust correlation between rhythm disorders and the onset of depression.^[^ ^3^, ^4^, ^5^ ^]^ Laboratory studies indicate that aberrant light stimulation in mice can lead to cognitive impairment and depressive symptoms through the retina–brain neural pathway.^[^ ^6^ ^]^ Additionally, knockdown of the key rhythm gene Brain and Muscle ARNT‐Like 1 (*BMAL1)* disrupts circadian rhythms within the suprachiasmatic nucleus and elevates depressive‐like behaviors in mice.^[^ ^7^ ^]^ Collectively, these studies suggest that CRD may contribute to depressive episodes. However, the majority of existing research on the relationship between CRD and depression has been primarily correlational, and the specific mechanisms through which CRD induces or exacerbates depression remain unknown.

The *Period* (Per) gene, a crucial component in the regulation of circadian rhythms, can influence clock‐controlled genes through the negative regulation of core clock genes Circadian Locomotor Output Cycles Kaput (*CLOCK) and BMAL1*, thereby affecting mitochondrial function, energy metabolism, and the redox state of cells. Numerous studies on the expression of Per genes have demonstrated that Per proteins play significant roles in regulating various physiological processes and cellular functions, including lipid metabolism,^[^ ^8^ ^]^ mitochondrial function,^[^ ^9^ ^]^ and stem cell differentiation.^[^ ^10^ ^]^ When circadian rhythm is disrupted, abnormal expression of rhythm genes occurs in both central to peripheral cells, leading to alterations in the physiological processes and cellular functions they regulate. This disruption may promote the onset of pathological processes associated with CRD. Research has demonstrated that the simultaneous knockdown of *Period 1* (*Per1*) and *Period 2* (*Per2*) rhythm genes in the nucleus accumbens increases anxiety‐like responses in mice.^[^ ^11^ ^]^ However, it remains unclear whether Per gene expression is aberrant in peripheral tissues and organs during the development of depression induced by CRD, and what role it plays in the pathogenesis of depression.

The gut microbiota represents the largest and most complex microecosystem in the human body. Gut microbes and their metabolites play a crucial role in regulating the physiological functions of the body and maintaining overall health.^[^ ^12^, ^13^ ^]^ A substantial body of evidence indicates that the gut bacterial community is integral to the development of depression; specifically, the composition and abundance of gut bacteria are significantly altered in depressed patients and animal models compared to controls.^[^ ^14^, ^15^ ^]^ Furthermore, the transplantation of gut bacteria from depressed patients or animal models to normal animals has been shown to induce a depressive behavioral phenotype.^[^ ^12^, ^16^ ^]^ Intensive mechanistic studies suggest that gut microbiota may contribute to the development of depression by influencing the central nervous system through direct stimulation of the vagus nerve, modulating systemic inflammation via interactions with intestinal immune cells, and releasing altered metabolites and active substances.^[^ ^17^ ^]^ However, the relationship between key etiological factors of depression and the development of gut microbiota remains incompletely understood, particularly regarding the potential involvement of changes in peripheral rhythmic genes. In this study, we reveal a new mechanism that the disruption of intestinal epithelial *Per2* expression rhythm in CRD mice induces gut barrier damage, disturbance of gut microbiota and metabolites, peripheral and central inflammation, deficit in hippocampal neurogenesis, and impairment of synaptic function, thus contributing to the development of depression.

## 2 Results

### 2.1 Circadian Rhythm Disruption Induces Depression‐Like Phenotypes, Disturbed Expression of Intestinal Epithelial *Per2* Gene, and Gut Microbiota Dysbiosis in Mice

**Figure 1:**
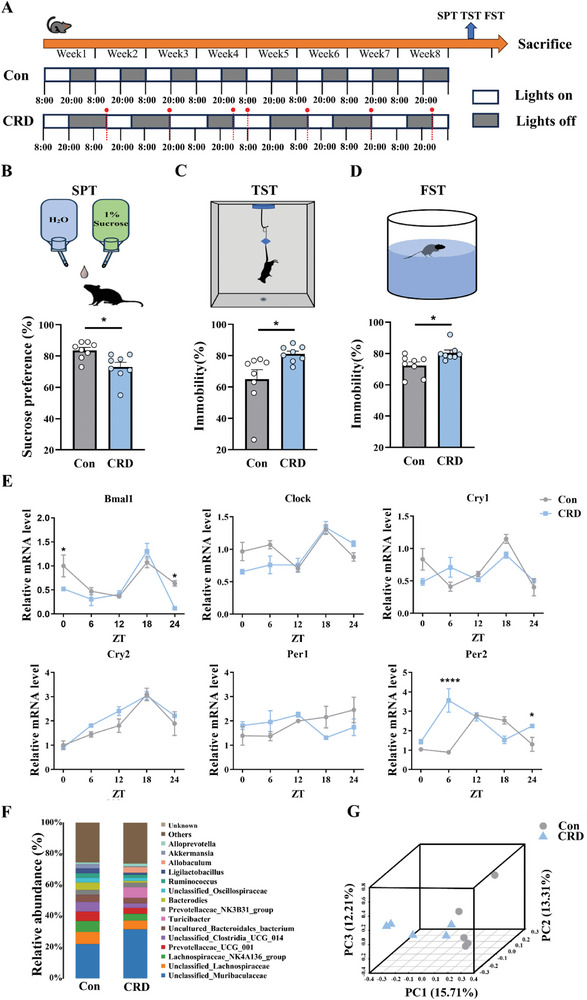
Circadian rhythm disruption induces disturbed expression of intestinal epithelial *Per2* gene, gut microbiota dysbiosis, and depression‐like phenotypes in mice. A) Schematic diagram illustrating the establishment of mouse model with circadian rhythm disruption (CRD). Two months old male C57BL/6 mice were exposed to weekly 6 h backward phase shifts of the light and dark (LD) cycle for 8 weeks, then behavior tests were performed before their sacrifice. B) The schematic diagram of sucrose preference test (SPT) and the sucrose preference ratio (%) of the mice, *n* = 8 for each group. C) The schematic diagram of tail suspension test (TST) and the immobility time ratio (%) of the mice, *n* = 8 for each group. D) The schematic diagram of forced swimming test (FST) and the immobility time ratio (%) of the mice, *n* = 8 for each group. E) Relative expression of core oscillator and clock‐controlled genes in colon tissue samples collected at five time points across the 12 h L/D cycle. F) Relative abundance of gut microbiota operational taxonomic units (OTUs) assigned at the genus level in two groups. G) Unsupervised 3D principal component analysis (3D‐PCA) of gut microbiota. Shown in parentheses are the percentages of variation explained by each principal component (PC) between the two groups. CRD, circadian rhythm disruption; OTUs, operational taxonomic units; SPT, sucrose preference test; TST, tail suspension test; FST, forced swimming test. Data are represented as mean ± standard error of the mean (SEM). *p < 0.05, ****p < 0.0001.

To investigate whether CRD promotes the development of depression, we first established a CRD mouse model through subjecting the mice to a weekly 6 h light–dark (LD) cycle of backward shifting for 2 months (**Figure** **1**A). The successful establishment of the model was confirmed by electroencephalogram (EEG) detection^[^ ^18^ ^]^ (Figure S1A–C, Supporting Information) and rectal temperature changes (Figure S1D, Supporting Information). The CRD mice exhibited significantly different power spectral density changes and rectal temperatures compared to the control group. At the end of the intervention, classic behavior tests of depression in rodents including sucrose preference test (SPT), tail suspension test (TST), and forced swimming test (FST) were conducted to evaluate the depressive states of the CRD mice. The results showed that the CRD mice exhibited reduced sucrose intake compared to the control mice, indicating anhedonia (Figure 1B). In both TST and FST, CRD mice displayed an increased immobility time ratio compared to the control group (Figure 1C,D), indicating the presence of despairing behaviors in mice. Additionally, analysis of the intestinal epithelial rhythm genes revealed an abnormal expression of the *Per2* gene, which showed a complete loss of normal rhythmicity (Figure 1E). Previous studies have documented that CRD increases intestinal epithelial barrier permeability and leads to gut microbiota dysbiosis in mice.^[^ ^19^ ^]^ In our study, comparative analysis of bacterial abundance and structure between the two groups revealed significant alterations (Figure 1F). A decrease in the abundance of *Lachnospiraceae, Prevotellaceae_UCG_001*, and *Bacteroides* was observed, along with an increase in *Muribaculaceae, Turicibacter*, and *Allobaculum*. 3D principal component analysis (PCA) analysis also illustrated a clear distinction between the two groups (Figure 1G). Furthermore, Spearman's correlation analysis between the abundances of the differentially abundant taxa and the outcomes of three classical depressive behavioral paradigms (SPT, TST, and FST) showed that the vast majority of these microbial changes (at both the phylum and genus levels) were robustly correlated with the severity of depressive‐like behaviors (Figure S2, Supporting Information). Collectively, these findings suggest that disruption of circadian rhythm alters the expression of intestinal epithelial *Per2* gene, leads to gut microbiota dysbiosis, and induces depression‐like phenotypes in mice; and CRD‐related gut microbiota dysbiosis may contribute to the development of depression.

### 2.2 Specific Knockout of *Per2* in Intestinal Epithelium Is Sufficient to Prevent CRD‐Induced Depression, Impairment of Intestinal Barrier, and Dysbiosis of Gut Microbiota

**Figure 2:**
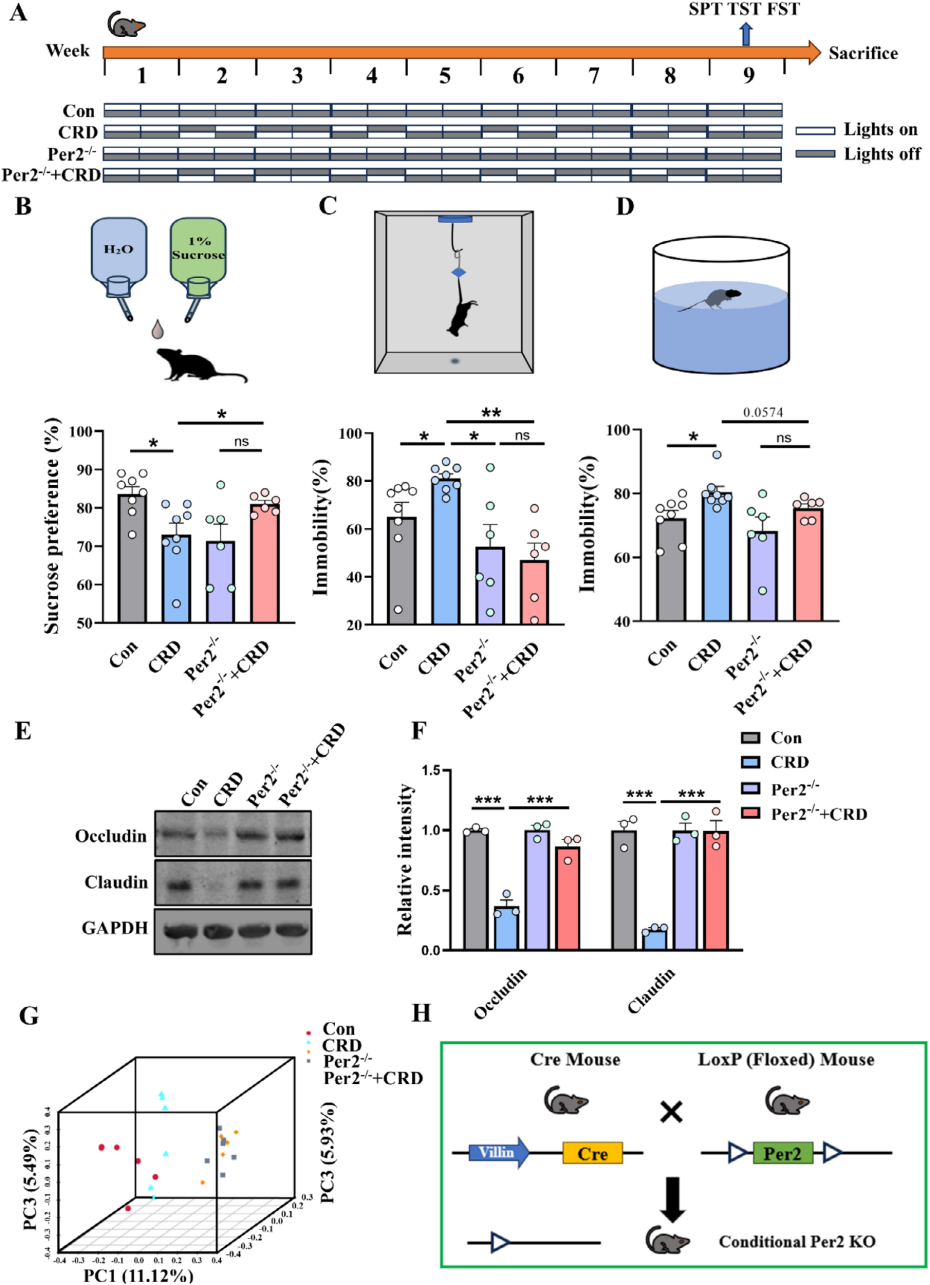
Specific deletion of intestinal epithelial *Per2* prevents CRD‐induced depression, alleviates impairment of intestinal barrier and gut microbiota dysbiosis. A) Schematic diagram illustrating the experimental design. B) The schematic diagram of SPT and the sucrose preference ratio (%) of the mice, *n* = 8 for Con and CRD groups, *n* = 6 for Per^−/−^ and Per^−/−^ + CRD groups. C) The schematic diagram of TST and the immobility time ratio (%) of the mice, *n* = 8 for Con and CRD groups, *n =* 6 for Per^−/−^ and Per^−/−^ + CRD groups. D) The schematic diagram of FST and the immobility time ratio (%) of the mice, *n* = 8 for Con and CRD groups, *n* = 6 for Per^−/−^ and Per^−/−^ + CRD groups. E,F) Representative immunoblots and quantitative analysis of intestinal barrier protein Occludin and Claudin in colon tissue homogenates, *n* = 3 for each group. G) Unsupervised 3D‐PCA of gut microbiota. Shown in parentheses are the percentages of variation explained by each PC in the four groups *n* = 5 for each group. H) Construction of *Per2* intestinal epithelial conditional knockout mice using Cre‐LoxP system. CRD, circadian rhythm disruption. Data are represented as mean ± SEM. *p < 0.05, **p < 0.01, ***p<0.001, ns: no significance.

The rhythmic disruption of intestinal epithelial *Per2* gene indicates a possible involvement of this gene in CRD‐induced disturbances, we thus explored whether intestinal epithelial‐specific *Per2* deletion influences the CRD‐induced depressive phenotype. To this end, we generated intestinal epithelial‐specific *Per2* knockout mice (*Per2* ^−/−^) by crossing *Per2* ^fl/fl^ mice with Villin1^Cre^ mice. The specific ablation of *Per2* protein exclusively in the intestinal epithelium was confirmed by Western blotting and immunofluorescence (Figure S3, Supporting Information). Subsequently, these *Per2* ^−/−^ mice and their control littermates were subjected to the CRD procedure (**Figure** **2**A,H). Notably, deletion of *Per2* in intestinal epithelial cells effectively prevented the CRD‐induced depressive‐like phenotype, as evidenced by the restoration of SPT (Figure 2B) and a decrease in the immobility time ratio (%) measured by the TST and FST (Figure 2C,D). There were no changes in locomotor ability among the four groups of mice, as indicated by the lack of significant differences in total distance and average speeds (Figure S4A,B, Supporting Information). Tight junction proteins are crucial molecules for maintaining the integrity of intestinal barrier, and a lower protein level of the tight junction protein Occludin is associated with increased intestinal leakiness.^[^ ^19^ ^]^ We subsequently examined intestinal barrier proteins and found that the expression of Occludin and Claudin significantly decreased in the colon of CRD mice, and this decrement was not observed in *Per2* ^−/−^ + CRD mice when comparing to *Per2* ^−/−^ mice (Figure 2E,F and Figure S5 (Supporting Information)). Concurrently, *Per2* deletion largely modified the overall structure of the gut microbiota and mitigated the CRD‐induced alterations. The gut microbiota structure was comparable between *Per2* ^−/−^ and *Per2* ^−/−^ + CRD mice (Figure 2G). Collectively, these results suggest that intestinal epithelial deletion of *Per2* is sufficient to prevent the CRD‐induced depressive phenotype, as well as the associated barrier impairment and gut microbiota dysbiosis.

### 2.3 Specific Deletion of Intestinal Epithelial *Per2* Prevents CRD‐Induced Blood–Brain Barrier Damage and Neuroinflammation, Rescues Neurogenesis and Synaptic Function in the Hippocampus of Mice

**Figure 3:**
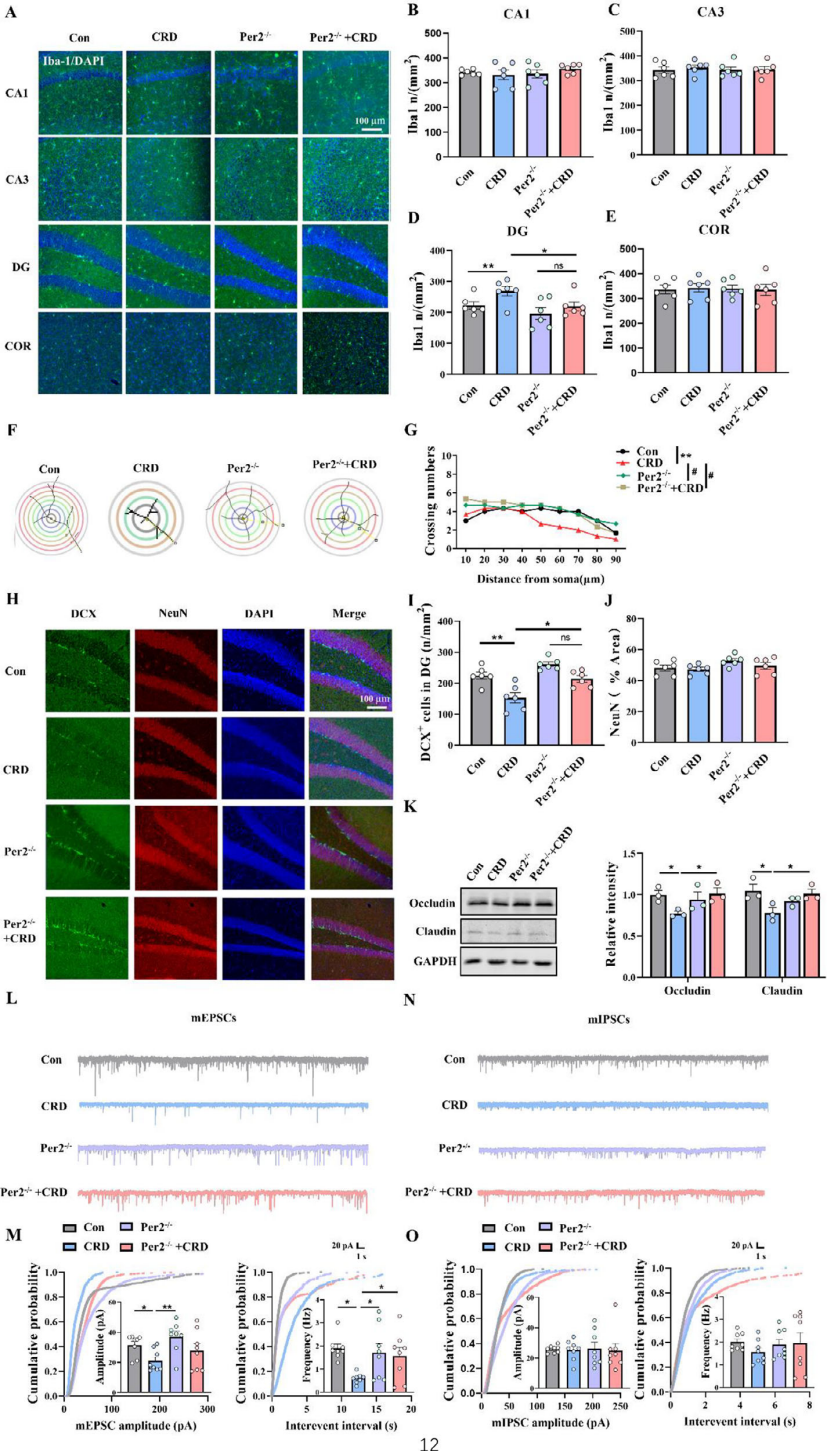
Specific deletion of intestinal epithelial *Per2* prevents CRD‐induced blood–brain barrier damage, neuroinflammation, and hippocampal neurogenesis impairment, restoring neuronal function in mice. A) Representative images of Iba‐1 immunofluorescence in different regions of the hippocampus and cortex. Scale bar: 100 µm. B–E) Quantification of Iba‐1‐positive microglia number in the four groups of mice, *n* = 6 for each group. F) Scheme of Sholl analysis of a skeletonized microglia with branch and superimposed centric circles. G) Sholl analysis of dendritic arborization of microglia in (F). H) Representative immunostaining images with antibodies against DCX (green) and NeuN (red) to show the newborn unmatured neurons and mature neurons in the hippocampal DG, counterstained with DAPI (blue), *n* = 6 for each group. Scale bar: 100 µm. I,J) Quantification of DCX^+^ cells and the number of the NeuN‐positive immunostaining area in (H), *n* = 6 for each group. K) Representative immunoblots and quantitative analysis of barrier protein Occludin and Claudin in hippocampal tissue homogenates from four groups of mice, *n* = 3 for each group. L) Representative miniature excitatory postsynaptic current (mEPSC) traces recorded in hippocampal DG. M) Cumulative distribution of mEPSC amplitude (left) and interevent intervals (right) (*n* = 8 cells from three mice). N) Representative miniature inhibitory postsynaptic current (mIPSC) traces recorded in hippocampal DG. O) Cumulative distribution of mIPSC amplitude (left) and interevent intervals (right) (*n* = 8 cells from three mice). CRD, circadian rhythm disruption; mEPSCs: miniature excitatory postsynaptic currents; mIPSCs: miniature inhibitory postsynaptic currents. Data are represented as mean ± SEM. *p < 0.05, **p < 0.01, ^#^ *p*<0.05, ns: no significance.

When epithelial barriers in the intestinal or respiratory tract are compromised, the integrity of other barriers, such as the blood–brain barrier (BBB), may also be disrupted. Defects in these barriers can lead to the recruitment and activation of immune cells, and subsequent inflammation.^[^ ^20^ ^]^ We thus investigated whether neuroinflammation and disruption of blood–brain barrier occurred in CRD mice. Microglia, the resident immune cells in the brain that regulate neuroinflammation, were examined for their morphology and characteristics. The number of microglia in the dentate gyrus (DG) region of the hippocampus significantly increased in CRD mice (**Figure** **3**A–E), with a typical morphology of activation (Figure 3F,G). Detection of blood–brain barrier proteins showed significant decreases in Occludin and Claudin levels in hippocampal tissues of CRD mice, indicating blood–brain barrier damage (Figure 3K). Notably, intestinal epithelial *Per2* deletion protected the mice from most of these above disruptions induced by CRD. Neuroinflammation has been shown to disrupt adult neurogenesis.^[^ ^21^ ^]^ To elucidate the mechanism underlying CRD‐induced depression, we identified newborn immature neurons through immunofluorescence staining with an antibody against Doublecortin (DCX) in the DG area. The results revealed that DCX‐labeled cells were significantly reduced in the hippocampal DG of CRD mice, whereas no significant difference in neurogenesis was observed between the *Per2* ^−/−^ + CRD and *Per2* ^−/−^ group (Figure 3H–J). These findings suggest that CRD‐induced neuroinflammation and deficits in neurogenesis may underlie the phenotype of depression, with the protective effect of *Per2* intestinal epithelial deletion potentially achieved, in part, by preserving normal BBB barrier function and neurogenesis.

Impaired synaptic transmission has been widely recognized as a cellular basis for the pathogenesis of depression.^[^ ^22^ ^]^ We further investigated whether CRD would affect excitatory and inhibitory synaptic transmission in the hippocampal DG region. To this end, we recorded miniature inhibitory postsynaptic currents (mIPSCs) and miniature excitatory postsynaptic currents (mEPSCs) in the DG using the whole‐cell patch‐clamp technique. Our results demonstrated a reduction in both the frequency and amplitude of mEPSCs (Figure 3L,M), while mIPSCs remained unaffected (Figure 3N,O) in the DG region of CRD mice. Notably, this impairment induced by CRD was alleviated in *Per2* ^−/−^ mice, suggesting that CRD may disrupt the function of excitatory synaptic transmission, which can be rescued by conditional knockout of intestinal epithelial *Per2*.

### 2.4 Transplantation of the CRD Gut Microbiota Induces Depressive‐Like Behaviors

**Figure 4:**
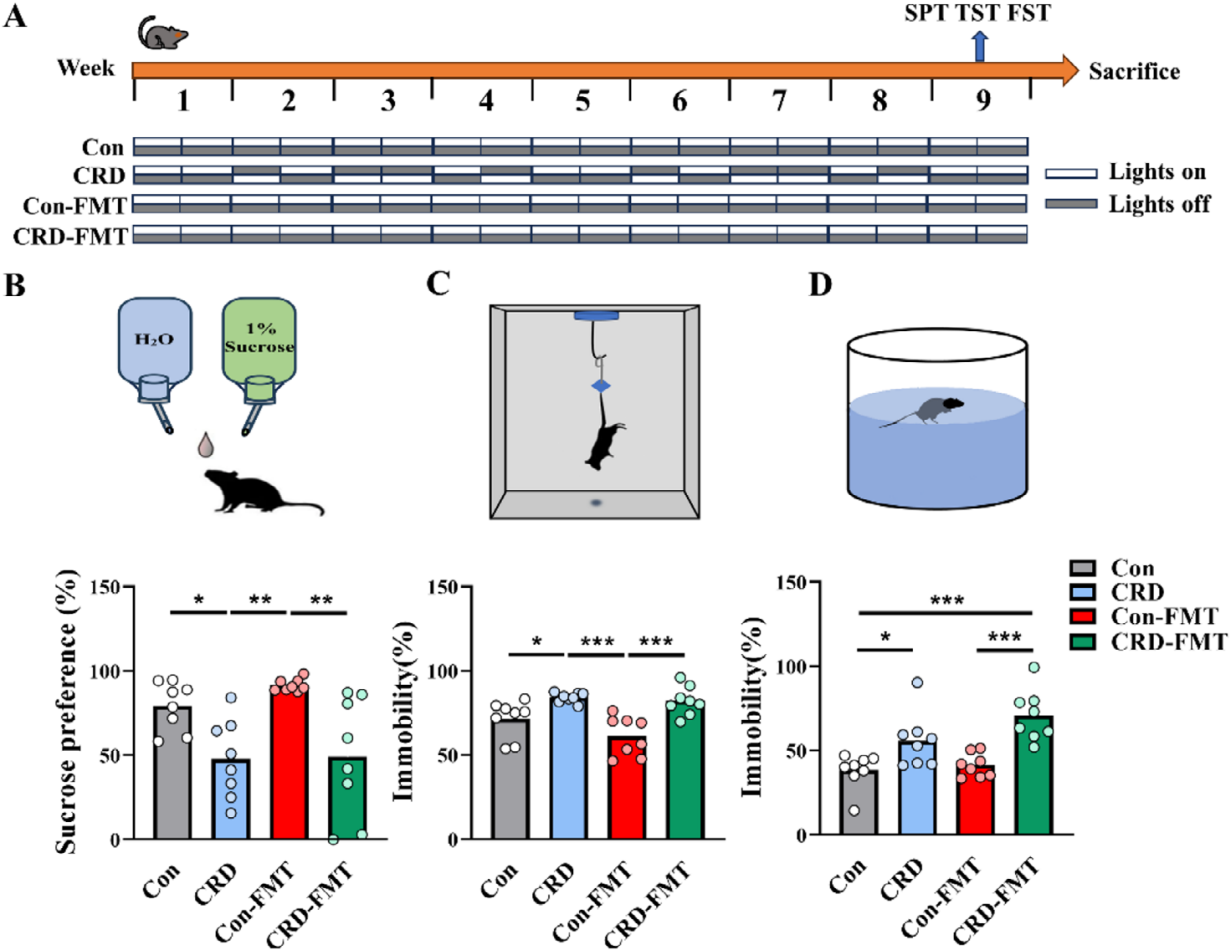
Gut microbiota transplantation from CRD to normal mice induces depression‐like phenotypes. A) Schematic diagram illustrating the experimental design of fecal microbiota transplantation (FMT) from control or CRD mice to mice which are removed of most gut bacteria by antibiotics treatment. B) The schematic diagram of SPT and the sucrose preference ratio (%) of the mice, *n* = 8 for each group. C) The schematic diagram of TST and the immobility time ratio (%) of the mice, *n* = 8 for each group. D) The schematic diagram of FST and the immobility time ratio (%) of the mice, *n* = 8 for each group; CRD, circadian rhythm disruption; Con‐FMT, transplantation of control fecal microbiota to normal mice. CRD‐FMT, transplantation of CRD fecal microbiota to normal mice. Data are represented as mean ± SEM. *p < 0.05, **p < 0.01, ***p < 0.001.

**Figure 5:**
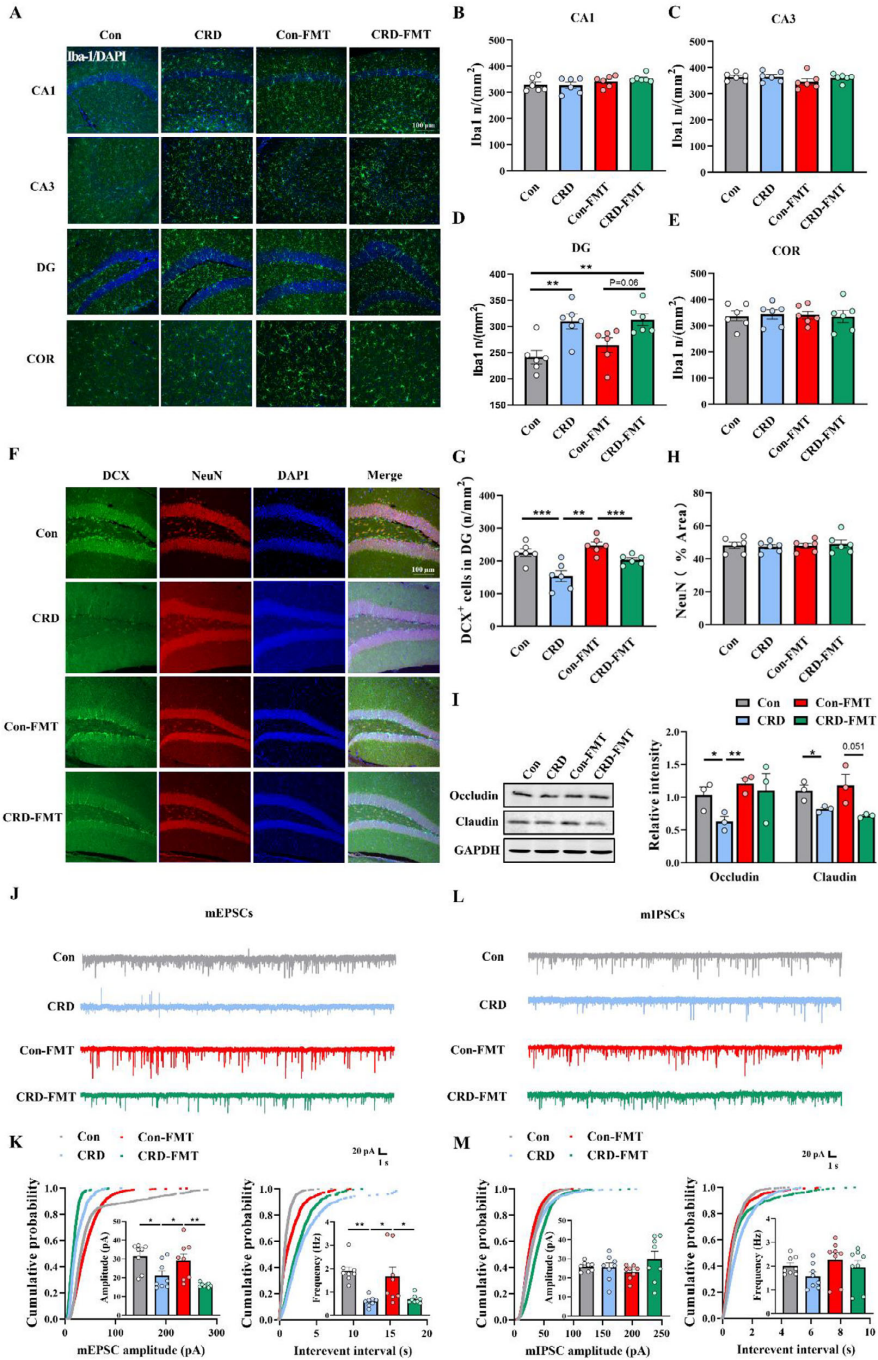
Gut microbiota transplantation from CRD to normal mice induces blood–brain barrier damage and neuroinflammation, impairs hippocampal neurogenesis and neurological function. A) Representative images of Iba‐1 immunofluorescence in different regions of the hippocampus and cortex. Scale bar: 100 µm. B–E) Quantification of Iba‐1‐positive microglia number in the four groups of mice, *n* = 6 for each group. F) Representative immunostaining images with antibodies against DCX (green) and NeuN (red) to show the newborn unmatured neurons and mature neurons in the hippocampal DG, counterstained with DAPI (blue). Scale bar: 100 µm. G,H) Quantification of the number of DCX^+^ cells and the NeuN‐positive immunostaining area in (F), *n* = 6 for each group. I) Representative immunoblots and quantitative analysis of barrier protein Occludin and Claudin in hippocampal tissue homogenates from four groups of mice, *n* = 3 for each group. J) Representative mEPSC traces recorded in hippocampal DG. K) Cumulative distribution of mEPSC amplitude (left) and interevent intervals (right) (*n* = 8 cells from three mice). L) Representative mIPSC traces recorded in hippocampal DG. M) Cumulative distribution of mIPSC amplitude (left) and interevent intervals (right) (*n* = 8 cells from three mice). CRD, circadian rhythm disruption; FMT, fecal microbiota transplantation; mEPSCs: miniature excitatory postsynaptic currents; mIPSCs: miniature inhibitory postsynaptic currents. Data are represented as mean ± SEM. *p < 0.05, **p < 0.01, ***p < 0.001.

To investigate whether changes in the gut microbiota contribute to behavioral alteration in CRD mice, we conducted fecal microbiota transplantation (FMT) experiments, as illustrated in **Figure** **4**A. Interestingly similar to CRD mice, the recipients of CRD microbiota exhibited a decreased sucrose preference in the SPT (Figure 4B) and an increased immobility time ratio (%) in the TST and FST (Figure 4C,D) comparing to con microbiota recipient mice. There were no changes in the locomotor ability among the four groups of mice (Figure S4C,D, Supporting Information). We further examined neuroinflammation and neurogenesis. The results indicated that the number of microglia in the DG region was significantly increased in both CRD and CRD microbiota recipient mice (**Figure** **5**A–E). Consistent with the changes in CRD mice, hippocampal barrier protein levels of Occludin and Claudin were reduced in mice transplanted with CRD microbiota (Figure 5I). Additionally, DCX‐labeled cells were significantly reduced in hippocampal DG of these groups (Figure 5F–H), and a reduction in both the frequency and amplitude of mEPSCs (Figure 5J,K) but not of mIPSCs (Figure 5L,M) in the DG region was observed. Both Con‐FMT and CRD‐FMT recipient mice were pretreated with antibiotics before the fecal microbiota transplantation. To rule out potential confounding effects of the antibiotics, a separate cohort of mice receiving antibiotics alone was subjected to a comprehensive assessment. The analysis revealed no evidence of compromised intestinal barrier integrity, neuroinflammation, impaired neurogenesis, or neuronal dysfunction (Figures S6 and S7, Supporting Information). Collectively, these results suggest that gut microbiota transplantation from CRD to normal mice induces blood–brain barrier damage and neuroinflammation, impairs hippocampal neurogenesis and neurological function. Gut microbiota dysbiosis induced by CRD plays a key role in mediating BBB damage, neuroinflammation, and subsequent deficits in neurogenesis and synaptic function.

Rifaximin is a nonabsorbable antibiotic, which can regulate the structure of the gut microbiome, protect intestinal barrier, and reduce gut‐derived inflammation.^[^ ^19^, ^23^ ^]^ To further identify that gut microbiota dysbiosis is upstream of above pathological changes, we treated CRD mice with rifaximin through gavage during the last week of model establishment. As demonstrated in Figure S8A (Supporting Information), rifaximin effectively mitigated the depression‐like phenotype resulting from rhythmic disturbance, as evidenced by SPT, FST, and TST results (Figure S8B–D, Supporting Information), accompanied by no significant change in motor ability among the four groups (Figure S8E,F, Supporting Information). At the same time, rifaximin effectively protected against CRD‐induced damage to both the gut barrier and blood–brain barrier, as well as subsequent central neuroinflammation, impaired neurogenesis, and functional impairment in the hippocampal DG region (Figures S8G,H and S9, Supporting Information).

### 2.5 CRD‐Induced *Per2*‐Dependent Gut Microbiota Dysbiosis Is Associated with Disturbed Tryptophan Metabolism and Systemic Inflammation

**Figure 6:**
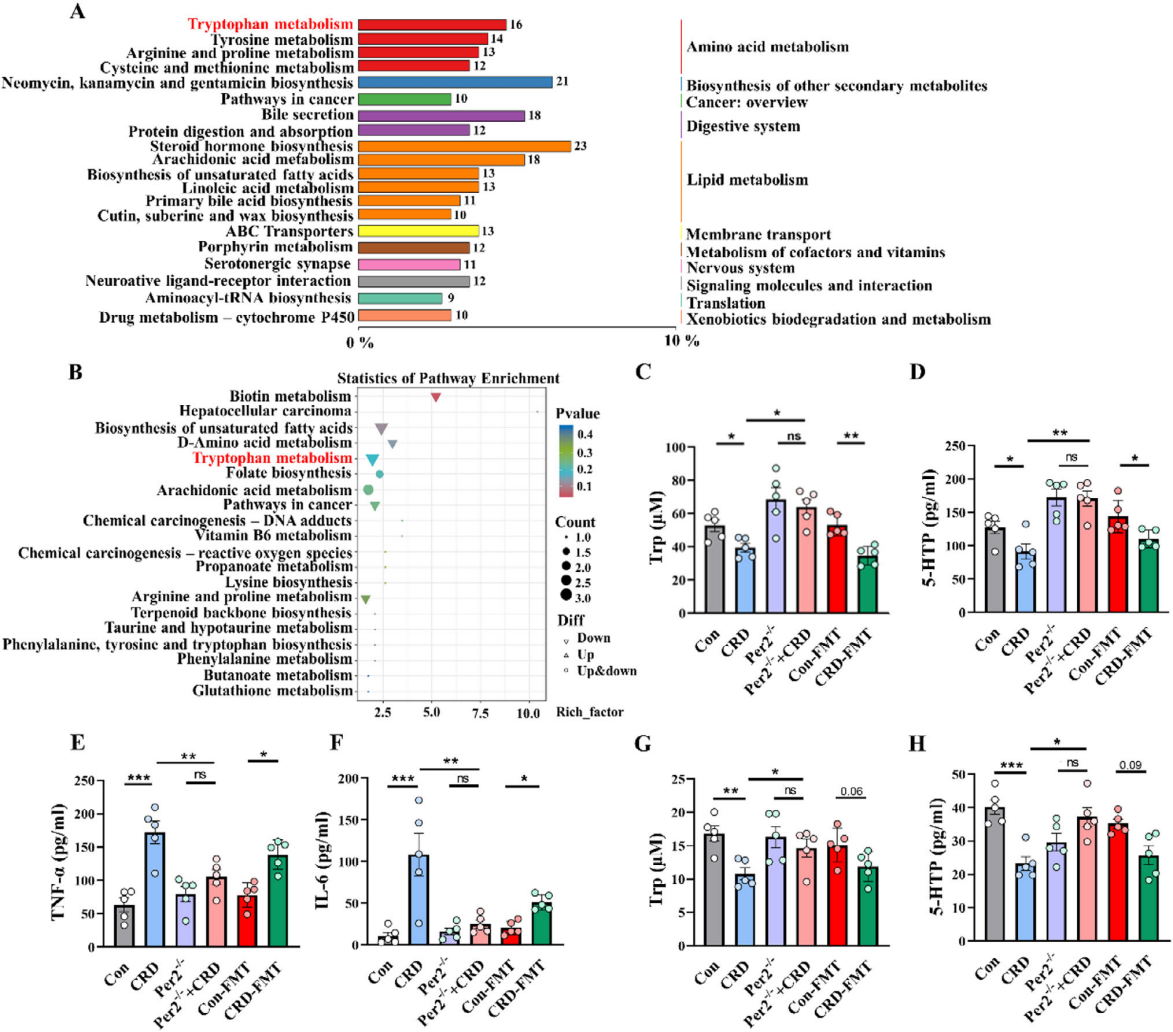
CRD‐induced *Per2‐*dependent gut microbiota dysbiosis is related with disturbed tryptophan metabolism and systemic inflammation. A) Characterization and quantification of fecal metabolites followed by data annotation analysis and functional enrichment in mice of control and CRD groups. Integrated analysis of KEGG pathways of gut microbiota metabolome showed the number of metabolites annotated, column length represents the number of significant metabolites annotated to this pathway, *n* = 5 for each group. B) Differential metabolite KEGG enrichment maps, up/downregulated differential metabolites are indicated by up/down triangles, and metabolic pathways that contain both up‐ and downregulated metabolites are indicated by circles, *n* = 5 for each group. C,D) Quantification of Trp and 5‐HTP levels in serum of mice in Con, CRD, *Per2* ^−/−^, *Per2* ^−/−^ + CRD, Con‐FMT, and CRD‐FMT groups, *n* = 5 for each group. E,F) Quantification of TNF‐α and IL‐6 levels in serum of different groups of mice, *n* = 5 for each group. G,H) Quantification of Trp and 5‐HTP levels in hippocampal tissues of different groups of mice, *n* = 5 for each group, CRD, circadian rhythm disruption; FMT, fecal microbiota transplantation. Data are represented as mean ± SEM. *p < 0.05, **p < 0.01, ***p < 0.001, ns: no significance.

Gut microbiota exerts potent regulation on physiological function of host through multiple pathways, one of which is metabolites produced or modified by bacteria.^[^ ^12^ ^]^ To investigate how gut microbiota remodeling in CRD mice causes neuroinflammation and impairments in neurogenesis and synaptic function, fecal metabolome profiling was performed at the end of two months of CRD. The metabolome analysis indicated that significant metabolites from the gut microbiota of CRD mice was predominantly enriched in lipid and amino acid metabolism, with tryptophan metabolism being particularly downregulated compared to that of control mice (**Figure** **6**A,B), this indicates a downregulation of the tryptophan‐related pathway in CRD‐exposed mice. Meanwhile, to elucidate the functional ramifications of the CRD‐induced dysbiosis, we conducted a predictive metagenomic analysis using Phylogenetic Investigation of Communities by Reconstruction of Unobserved States 2. This approach pinpointed that pathways pertaining to tryptophan metabolism were the most prominent functional signature distinguishing the microbial profile of the CRD group (Figure S10A,B, Supporting Information). Subsequently, we examined the levels of tryptophan and 5‐hydroxytryptophan (5‐HTP) in serum. Consistent with the metabolome analysis results, a significant decrease in tryptophan and 5‐HTP levels in the serum of CRD mice and mice transplanted with CRD microbiota was observed (Figure 6C,D), and Spearman's correlation analysis showed a significant correlation between metabolites (tryptophan (Trp) and 5‐HTP) and the differential microbiota (Figure S10C, Supporting Information). An increase in serum Tumor Necrosis Factor‐alpha (TNF‐α) and interleukin‐6 (IL‐6) levels (Figure 6E,F) was also observed in CRD mice and mice transplanted with CRD microbiota. Detection of tryptophan and 5‐HTP levels in the hippocampus revealed alterations similar to those observed in serum (Figure 6G,H). Notably, intestinal epithelial *Per2* deletion mitigated these changes. Taking together, these results strongly suggest that in CRD mice, disturbed *Per2* expression in intestinal epithelium causes intestinal barrier damage and gut microbiota dysbiosis, the latter, contributes to the downregulation of tryptophan and 5‐HTP in serum and brain, which may highly correlate to systemic/central inflammation and neuronal dysfunction.

### 2.6 Tryptophan Supplementation Prevents CRD‐Induced Depression, Improves Tryptophan Metabolism, Reduces Systemic and Central Inflammatory Response, and Rescues Neurogenesis and Synaptic Function

**Figure 7:**
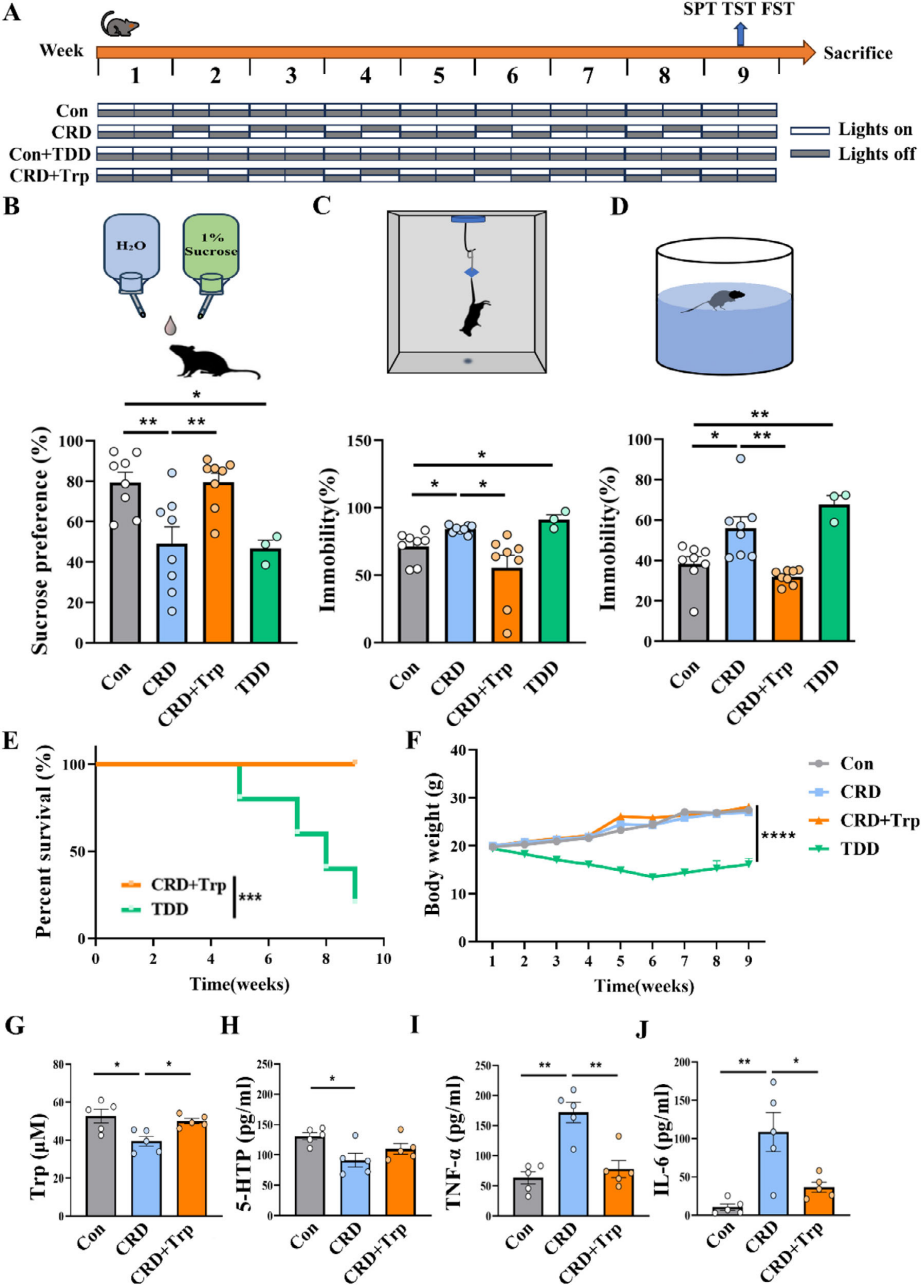
Tryptophan supplementation prevents CRD‐induced depression, increases tryptophan and 5‐HTP levels in serum, and reduces systemic inflammatory response in mice. A) Schematic diagram illustrating the experimental design: control mice were fed with tryptophan‐depleted diet (TDD), and CRD mice were fed with diet containing 0.4% tryptophan (CRD + Trp) for 9 weeks. B) The schematic diagram of SPT and the sucrose preference ratio (%) of the mice, *n* = 8 for Con, CRD, and CRD + Trp groups, *n* = 3 for TDD group. C) The schematic diagram of TST) and the immobility time ratio (%) of the mice, *n* = 8 for Con, CRD, and CRD + Trp groups, *n* = 3 for TDD group. D) The schematic diagram of FST and the immobility time ratio (%) of the mice, *n* = 8 for Con, CRD, and CRD + Trp groups, *n* = 3 for TDD group. E) Survival curves of the mice in CRD + Trp and TDD groups, *n* = 8 for each group. F) Body weight curves of the mice in four groups, *n* = 8 for each group. G,H) Quantification of Trp and 5‐HTP levels in serum of different groups of mice, *n* = 5 for each group. I,J) Quantification of TNF‐α and IL‐6 levels in serum of different groups of mice, *n* = 5 for each group. CRD, circadian rhythm disruption; TDD, Trp‐depleted diet; Trp, tryptophan. Data are represented as mean ± SEM. *p < 0.05, **p < 0.01, ***p < 0.001, ****p<0.0001.

To further confirm the key role of disturbed tryptophan metabolism in mediating the CRD‐induced pathological changes and depressive‐like phenotype, as illustrated in **Figure** **7**A, we tested the effects of tryptophan depletion in normal mice and tryptophan supplementation in CRD mice. The results showed that tryptophan supplementation effectively reverses the CRD‐induced depressive phenotype, as evidenced by an increased sucrose preference in the SPT (Figure 7B) and decreased immobility time ratio (%) in both the TST and FST (Figure 7C,D) in the CRD + Trp group compared to the CRD group. There were no significant changes in the locomotor ability among the four groups of mice (Figure S4E,F, Supporting Information). On the contrary, tryptophan depletion severely impacted the survival and health status of the mice, as evidenced by a mortality rate of up to 75% (Figure 7E) and only half of the body weight of surviving mice relative to normal mice at 9 weeks of age (Figure 7F). This may be attributed to the fact that tryptophan is an essential amino acid that cannot be synthesized by the body, further underscoring its significance for host health. We detected the depressive phenotypes in the surviving mice at the end of 9 weeks tryptophan depletion, and found that these mice exhibited significant depressive‐like behaviors compared with controls. These results reinforce the role of tryptophan metabolism defect in mediating depression in CRD mice. We subsequently examined serum levels of tryptophan and 5‐HTP and found that tryptophan supplementation mitigated the decrease in both tryptophan and 5‐HTP levels (Figure 7G,H) as well as the elevation of the inflammatory factor TNF‐α and IL‐6 (Figure 7I,J) induced by CRD.

**Figure 8:**
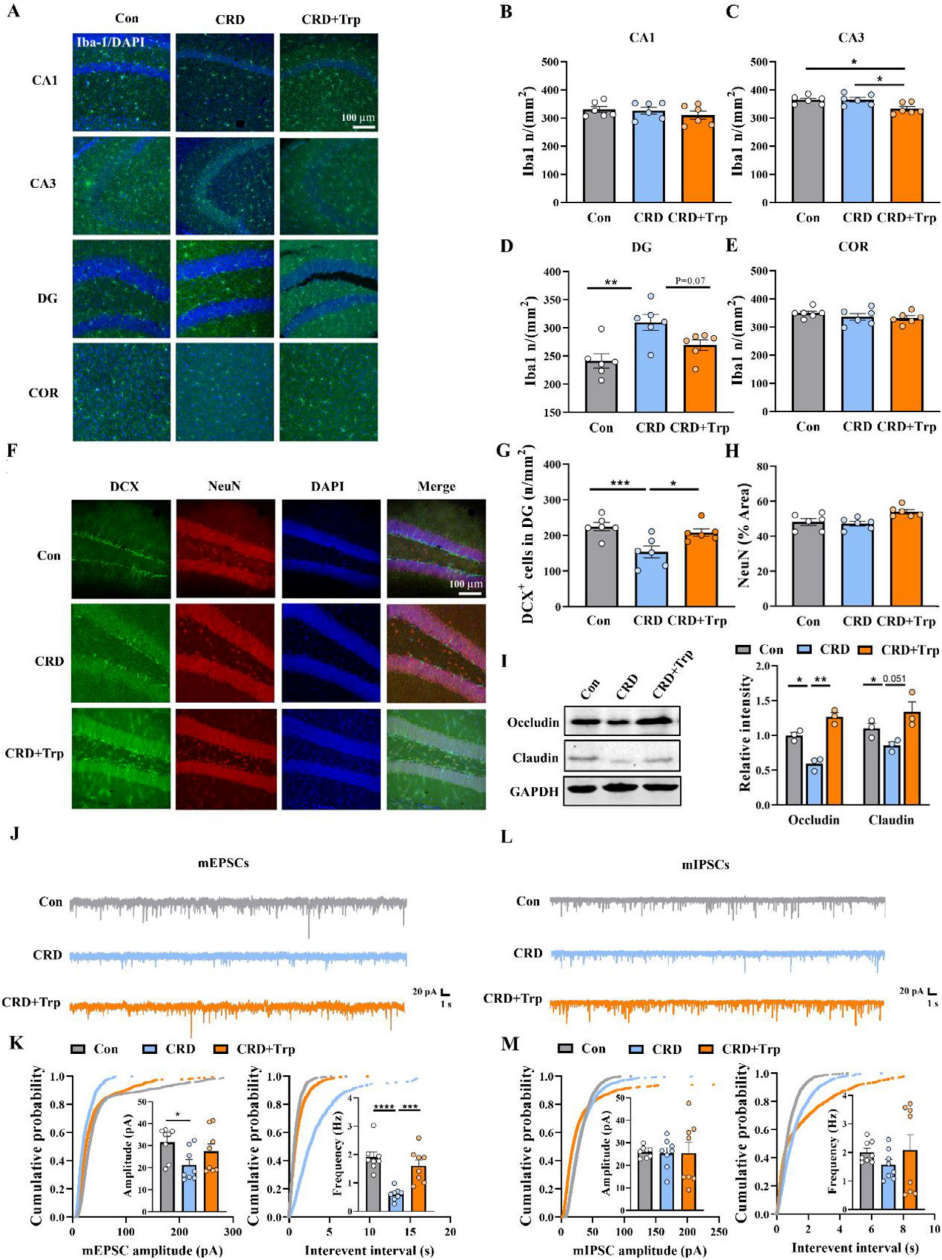
Tryptophan supplementation prevents CRD‐induced blood–brain barrier damage and neuroinflammation, protects hippocampal neurogenesis and neurological function. A) Representative images of Iba‐1 immunofluorescence in different regions of the hippocampus and cortex. Scale bar: 100 µm. B–E) Quantification of Iba‐1‐positive microglia number in three groups of mice, *n* = 6 for each group. F) Representative immunostaining images with antibodies against DCX (green) and NeuN (red) to show the newborn unmatured neurons and mature neurons in the hippocampal DG, counterstained with DAPI (blue). Scale bar: 100 µm. G,H) Quantification of the number of DCX^+^ cells and the NeuN‐positive immunostaining area in (F), *n* = 6 for each group. I) Representative immunoblots and quantitative analysis of barrier protein Occludin and Claudin in hippocampal tissue homogenates from the four groups of mice, *n* = 3 for each group. J) Representative mEPSC traces recorded in hippocampal DG. K) Cumulative distribution of mEPSC amplitude (left) and interevent intervals (right) (*n* = 8 cells from three mice). L) Representative mIPSC traces recorded in hippocampal DG. M) Cumulative distribution of mIPSC amplitude (left) and interevent intervals (right) (*n* = 8 cells from three mice). CRD, circadian rhythm disruption; Trp, tryptophan; mEPSCs: miniature excitatory postsynaptic currents; mIPSCs: miniature inhibitory postsynaptic currents. Data are represented as mean ± SEM. *p < 0.05, **p < 0.01, ***p < 0.001, ****p < 0.0001.

Next, we explored whether tryptophan intervention could reverse the pathological changes in the central nervous system induced by CRD. Examination of central inflammation revealed that the significant increase in the number of microglia in the DG area, induced by CRD, was reversed in the CRD + Trp group (**Figure** **8**A–E). Consistent with these findings, direct inhibition of active microglia by tryptophan, which was manifested by change in cell morphology and decreases in proinflammatory cytokines, was observed in lipopolysaccharide (LPS)‐stimulated microglia (Figure S11, Supporting Information). These data suggest that tryptophan deficiency may play a key role in CRD‐induced neuroinflammation. With the inhibition of neuroinflammation, the impairment of neurogenesis, as indicated by decreased number of DCX^+^ cells in hippocampal DG area (Figure 8F–H), and the impairment of BBB integrity, as indicated by decreased barrier protein Occludin and Claudin, were also rescued by tryptophan supplementation (Figure 8I). At last, mice in CRD + Trp group showed relatively normal frequency/amplitude of mEPSCs and mIPSCs (Figure 8J–M), indicating a rescue of neuronal function. These results collectively suggest that tryptophan supplementation is an effective strategy to ameliorate blood–brain barrier damage and neuroinflammation, thereby restoring hippocampal neurogenesis and neurological function, as well as the depressive phenotype induced by CRD.

## 3 Discussion

CRD has been identified associated with aging, immunocompromise, metabolic disorders, and mood disorders.^[^ ^24^, ^25^ ^]^ The causal relationship between CRD and depression has been long investigated, with the main focus on the role of abnormal rhythmic gene expression in certain brain regions, which may contribute to the onset of depression by disturbing neuronal activity, regulating metabolism, and other pathways.^[^ ^26^, ^27^ ^]^ Peripheral tissues and cells also express rhythmic genes, which are normally expressed in response to central clock, oscillating in cycles close to 24 h. However, it remains unclear whether rhythmic genes expressed in peripheral cells also contribute to the development of central nervous system disorders, particularly depression associated with CRD. In this study, we address this gap by revealing the strong correlation of abnormal *Per2* gene expression in intestinal epithelium with the dysregulated gut–brain–cerebral axis in CRD mice with depressive phenotype. To our knowledge, this is the first report showing participation of peripheral rhythmic gene in the development of depression.

Based on the numerous published studies which show that gut microbiota dysbiosis plays a crucial role in the development of depression,^[^ ^28^, ^29^ ^]^ we aimed our investigation at intestinal tract and discovered that the rhythmic homeostasis of peripheral intestinal epithelial cells is compromised due to CRD, with *Per2* showing the most significant alteration. Intervention experiments targeting *Per2* in intestinal epithelial cells confirmed its pivotal role, indicating that disturbances in peripheral rhythmicity contribute to the pathogenesis of depression linked to disruption in circadian system. For the mechanism of intestinal *Per2*‐dependent occurrence of depression in CRD, we identify that loss of normal *Per2* expression rhythm in epithelium results in gut barrier damage and gut microbiota disturbance, and these two events contribute to subsequent peripheral/central inflammatory responses, impaired neurogenesis, and synaptic dysfunction. The key role of gut barrier damage and gut microbiota disturbance in mediating the pathogenesis related to depression is further confirmed in FMT experiment in which CRD gut microbiota was transplanted to normal mice, and in mice which were treated with rifaximin to protect the gut barrier and prevent gut microbiota disturbance induced by CRD.

In our study, neuroinflammation, defect in neurogenesis, and a decrease in excitatory synaptic transmission are observed underlying the depressive phenotype in CRD mice. These results are consistent with previous studies performed on other depression models. Multiple studies have identified NLRP3‐mediated neuroinflammation as a key contributor to neuronal damage and the development of depression;^[^ ^30^, ^31^, ^32^ ^]^ the neurogenic hypothesis suggests that MDD is associated with neurogenic damage in the hippocampal DG, and that the antidepressant effects of fluoxetine are mediated through increased neurogenesis, whereas inhibition of neuronal excitability in newborn neurons would counteract the antidepressant effects of fluoxetine.^[^ ^33^, ^34^ ^]^ On the other hand, excitatory glutamatergic neuronal dysfunction has also been found to be a possible pathologic mechanism for the development of depression.^[^ ^35^ ^]^ The above identified pathophysiological cascade—encompassing neuroinflammatory activation, synaptic plasticity impairment, and gut–brain axis dysregulation—mechanistically underpins CRD‐induced depression.

To further fill the gap between gut microbiota disturbance and pathological changes related to depression such as neuroinflammation and neurological dysfunction in CRD, we analyzed gut microbiota composition and observed a significant decrease in the abundance of *Lachnospiraceae*, *Prevotellaceae_UCG_001*, and *Bacteroides*, alongside an increase in *Muribaculaceae*, *Turicibacter*, *Erysipelatoclostridium*, *Desulfovibrio*, and *Allobaculum*. These findings align with prior studies associating these genera with depression.^[^ ^36^, ^37^, ^38^, ^39^ ^]^ Furthermore, the strong correlation between the altered abundances of these taxa and the severity of depressive‐like behaviors supports a causative role of gut microbiota dysbiosis in CRD‐related depression. To further elucidate how these bacterial changes contribute to neurological dysfunction, we performed metabolomic analysis and found that CRD‐induced depression is mediated by disturbances in Trp metabolism. Decreased Trp and 5‐HTP levels were detected in the serum and brain of CRD mice and mice transplanted with CRD gut microbiota. It has been reported that some gut microorganisms, such as *Clostridium sporogenes*, *Peptostreptococcus anaerobius*, *Erysipelatoclostridium*, *Lactobacillus* spp, *Bifidobacterium* spp, *Bacteroides* spp, *Desulfovibrio, Clostridium* spp, and *Escherichia coli* directly transform Trp into several molecules, such as indole and its derivatives.^[^ ^40^, ^41^, ^42^, ^43^ ^]^ Of those members, *Erysipelatoclostridium* and *Desulfovibrio* showed increased abundance in CRD gut microbiota. These gut microorganisms might thus transform more tryptophan and decrease its absorption in the gut, reducing the plasma levels of tryptophan. Together with the decreased Trp and 5‐HTP in the serum and brain, peripheral and central inflammatory responses indicated by elevated levels of proinflammatory factors and microglial activation were observed in CRD mice and mice transplanted with CRD gut microbiota. And tryptophan supplementation is sufficient to elevate both Trp and 5‐HTP levels, reduce inflammatory responses, restore hippocampal neurogenesis and neurological function, and mitigate the depressive phenotype induced by CRD. The metabolism of tryptophan has a high participation in processes associated with the development of depression, such as dysregulation of neurotransmitters like serotonin (5‐HT) and neuroinflammation.^[^ ^44^ ^]^ In depression, the synthesis and metabolism of both 5‐HT and kynurenine, which are important neuroactive compounds derived from tryptophan in the body, are disturbed.^[^ ^45^ ^]^ 5‐HTP may help increase 5‐HT levels, reducing the symptoms of depression.^[^ ^46^ ^]^ On the other side, tryptophan and its metabolites may act as important immunomodulatory factors: the proinflammatory potential of the kynurenine metabolites^[^ ^47^, ^48^ ^]^ has been extensively reported; whereas 5‐methoxytryptophan, also a tryptophan metabolite, effectively inhibits microglia activation in response to LPS and reduces generation of inflammatory cytokines, and mitigates neuroinflammation in spinal cord injury.^[^ ^49^ ^]^ We also found that tryptophan incubation suppresses the LPS‐induced inflammatory transition in microglia. Another in vivo study reported that tryptophan supplementation promoted the proliferation and activation of Treg cells, thus suppressing the infiltration of CD8^+^ T cells into the brain and excessive activation of microglia, thereby ameliorating LPS‐induced cognitive impairment.^[^ ^50^ ^]^ Similar to our study, tryptophan‐rich diet alleviated inflammatory responses in brain of mouse model of chronic‐stress‐induced depression.^[^ ^51^ ^]^ Thus, tryptophan supplementation may exert protective effect in CRD mice through multiple pathways, including the modulation on 5‐HT synthesis and neuroinflammation. Taken together, disturbance in tryptophan metabolism plays a crucial role in the development of CRD‐induced depression and pathogenesis linked to the disease, and intervention on Trp directly through diet supplement is an effective strategy to treat the disease in mouse model.

In this study, we employed FMT experiments rather than using germ‐free mice to explore the correlation between CRD and the gut microbiota. This choice was made due to the limitations associated with germ‐free mice. Germ‐free mice have underdeveloped immune systems, abnormal gut morphology and functions, and high sensitivity to environmental factors.^[^ ^52^, ^53^ ^]^ These characteristics lead to microglial maturation deficits^[^ ^54^ ^]^ and synaptic developmental anomalies.^[^ ^55^ ^]^ By contrast, when normal mice are treated with antibiotics, although the number of gut microorganisms is reduced, a portion of the microbial community remains. These residual microorganisms can interact with the transplanted microbiota, mimicking a microbial ecological environment closer to the natural state. As a result, the experimental results are more clinically relevant and have greater translational potential.

While our findings offer a compelling mechanistic link between circadian disruption and depressive‐like behaviors, several limitations inherent to translating these preclinical results to human disease must be carefully considered. First, our model of circadian rhythm disruption, though robust and well‐established, primarily recapitulates the behavioral sequelae of physiological dysrhythmia. Human depression, in contrast, is a profoundly complex and heterogeneous disorder, with an etiology rooted in a multifaceted interplay of genetic predisposition, early life adversity, environmental exposures, and psychosocial stressors.^[^ ^56^ ^]^ Consequently, the linear pathogenic cascade we identified—from intestinal dysbiosis to a depressive phenotype—likely represents one of several potential etiologies within the broad clinical spectrum of MDD. Second, the mice gut microbiome, despite its functional analogies, differs substantially in composition from that of humans. The specific microbial taxa identified here as key regulators of tryptophan metabolism may not have direct orthologous counterparts in the human gut. Finally, while FMT served as a powerful tool to establish causality in our model, its therapeutic application for depression in clinical settings remains in its infancy. Significant challenges, including variable efficacy, long‐term safety concerns, and the need for rigorous standardization, must be overcome before it can be considered a viable treatment modality.

In conclusion, our study reveals that rhythm disorders can lead to the emergence of depressive phenotypes. We further identify that the disruption of the intestinal epithelial *Per2* rhythm gene accompanied by disturbance of gut microbiota and tryptophan metabolism, central inflammation, and impaired neurogenesis constitutes the intrinsic mechanisms underlying the development of depressive phenotypes. Despite these translational hurdles, our work charts a clear path for clinical investigation by identifying the intestinal circadian clock as a novel therapeutic target in depression. Future studies should now interrogate the expression of clock genes like in intestinal biopsies from MDD patients, particularly those with comorbid irritable bowel syndrome, and correlate these molecular signatures with intestinal permeability and tryptophan metabolism. This line of inquiry could pave the way for innovative chronotherapies—such as timed feeding or light exposure—to be tested as adjunctive treatments aimed at re‐entraining peripheral rhythms. Ultimately, our study provides a new mechanistic framework for gut–brain axis dysfunction in depression, shifting the focus toward peripheral circadian biology and offering a compelling rationale for developing microbiota‐ and metabolite‐targeted interventions.

## 4 Experimental Section

### Animals and CRD Modeling

C57BL/6J mice (male, 8 weeks, 22 ± 0.5 g) were purchased from the Experimental Animal Center of Tongji Medical College, Huazhong University of Science and Technology, and maintained under standard laboratory conditions (temperature: 22 ± 2 °C, humidity: 55 ± 5%) with unrestricted access to food and water. Mice in control groups had a consistent 12 h light:12 h dark cycle, with lights on at 8:00 AM and off at 8:00 PM daily. Circadian rhythm disruption was induced by implementing a weekly 6 h phase shift in the light and dark cycle over a period of 8 weeks.^[^ ^19^ ^]^ For rifaximin treatment, rifaximin (Sigma‐Aldrich) was dissolved in corn oil (37.5 g L^−1^) and administrated to the mice through gavage (250 mg kg^−1^ per day) daily in the last week of the experiment. The dosage and treatment duration were based on our previous study.^[^ ^19^ ^]^ Control mice were given the same volume of corn oil. The tryptophan dietary intervention was performed by giving feeds that removed tryptophan as well as feeds that contained 0.5% tryptophan throughout the whole experiment. All animal experiments were approved by the Animal Care and Use Committee of Huazhong University of Science and Technology (IACUC Number: 4651), and performed in compliance with the NIH Guide for the Care and Use of Laboratory Animals.

### EEG Recording

EEG signals were amplified using a Microelectrode AC amplifier model 1700 (A‐M Systems, USA) and digitized at a sampling rate of 500 Hz with a PCIe 6323 data acquisition board (National Instruments, USA). The recordings were conducted using Spikehound software (Neurobiological Instrumentation Engineer, USA). The raw EEG signals were analyzed using multitaper methods from the Chronux toolbox (version 2.1.2, Jarvisbio, Wuhan) within MATLAB 2016a (MathWorks, UK). Briefly, raw EEG data underwent band‐pass filtering (1–80 Hz) and band‐block filtering (48–52 Hz) to eliminate line noise. The analysis was conducted using a window size of 4 s (50% overlapping) within the frequency range of 0.5–50 Hz utilizing a 5‐taper fast Fourier transform. The average power spectral density and normalized power spectral density were calculated for the delta (0.5–4 Hz), theta (4–8 Hz), alpha (8–15 Hz), beta (15–25 Hz), and gamma (25–50 Hz) bands. The normalized power spectral density analysis was performed on the data from the control and CRD groups.

### Behavioral Tests—Open‐Field Test

In the open field test, the animals were placed in a 50 cm × 50 cm × 50 cm plastic container for 5 min. The floor of the container was divided into 25 sectors, arranged in a 5 × 5 grid. The central 3 × 3 sectors were designated as the “center area,” while the remaining sectors were classified as the “peripheral area.” The container was cleaned with 75% ethanol between each habituation period. The following parameters were recorded: total distance (mm) and average speed (mm s^−1^). All mice were acclimated in the testing room for at least for 2 h before the commencement of the testing session.

### SPT

In this experiment, mice were singly housed and acclimated to two bottles, one containing a 1% sucrose solution and the other containing drinking water for a duration of 12 h. The positions of the bottles were exchanged every 12 h to prevent the development of a position preference. Following a 12 h water deprivation period, the mice were exposed to the 1% sucrose solution and drinking water for another 12 h during the dark phase. Sucrose preference was calculated using the following formula: [sucrose consumption/(sucrose consumption + water consumption)] × 100%.

### TST

In the tail suspension test, the posterior third of the mouse's tail was secured with tape and suspended from a bracket, with the head positioned ≈15 cm above the table. All animals were suspended for a total of 5 min; during which the immobility time defined as the absence of any body or limb movements, aside from those associated with respiration was recorded in seconds during the final 5 min.

### FST

In the forced swim test, mice were individually placed into a transparent plastic container (18 cm in diameter and 25 cm in height) filled with water (25 ± 2 °C) to a depth of 15 cm, where they had no means of escape or contact with the bottom. The duration of immobility, defined as the time during which the animal did not exhibit escape responses, was recorded over a 5 min session. Subsequently, the animals were removed from the container and allowed to dry in a heated enclosure before being returned to their home cages. The cylinder was emptied, cleaned, and refilled with fresh water between each mouse.

### FMT

Recipient mice were given an antibiotic cocktail^[^ ^57^ ^]^ including vancomycin (0.5 g L^−1^), ampicillin (1 g L^−1^), neomycin (0.5 g L^−1^), and metronidazole (0.5 g L^−1^), administered in their drinking water for a duration of 7 consecutive days. All antibiotics were obtained from Sigma‐Aldrich. Fresh fecal transplants were pooled from Con and CRD donor mice, respectively. The homogenates were subsequently filtered through a 20 µm pore nylon filter to eliminate large particulate and fibrous matter. Following antibiotic treatment, the mice were orally challenged with 200 µL of fecal transplants (≈2 × 10^8^ viable probiotic bacteria dissolved in sterile phosphate‐buffered saline (PBS)) via gavage over a period of 28 consecutive days. The animals were colonized through multiple rounds of oral gavage with microbiota. Behavioral testing of the mice was conducted after the intervention.

### 16S rRNA Gene Sequence Analysis

Fresh fecal samples were collected at the end of the experiment for the preparation of a 16S rRNA gene amplicon library and subsequent sequencing to analyze gut microbiota. To assess bacterial diversity analysis, the V3–V4 variable regions of the 16S rRNA genes were amplified using universal primers 343F and 798R. The amplicon pools were prepared for sequencing, and the size and the quantity of the amplicon library were evaluated using a Qubit 3.0 and an Agilent 2100. Sequencing was conducted on Illumina's high‐throughput sequencing platform. Reads were screened for low‐quality bases and short read lengths, then assembled and assigned to operational taxonomic units with a similarity threshold of 97%. Alpha (α) and beta (β) diversity analyses were performed using the QIIME tool (version 1.9). Alpha diversity was assessed using the Chao 1 index to evaluate the complexity of microbial community composition within the samples. Beta diversity was determined using weighted UniFrac phylogenetic distance matrices, which were visualized through principal component analysis and analyzed via Analysis of Molecular Variance. The relative abundance of species at the genus level was presented to illustrate the community structure across different groups.

### Fecal Metabolic Profiling

The nontargeted metabolomics procedure was performed by electrospray ionization‐Q‐time of flight (TOF)/mass spectrometry (MS) (Xevo 121 G2‐S Q‐TOF, Waters) and UPLC‐QTOF/MS (ACQUITY UPLC I‐Class, Waters). Briefly, cecum samples (100 mg) were dissolved in 500 µL of ice‐cold water, mixed using a vortex, and centrifuged for 15 min at 12 000 rpm. Then, the supernatant was obtained, and the remaining precipitate was further extracted with 500 µL of ice‐cold methanol. The two fecal extracts were combined and centrifuged at 12 000 rpm for 15 min and the supernatant was stored at 4 °C; 10 µL of the supernatant was used for analysis. The MS data of cecum samples were first processed by MarkerLynx (version 4.1, Waters, Milford, MA, USA). The procedure included integration, normalization, and peak intensity alignment. In the positive data set, a list of *m*/*z* and retention time with corresponding intensities was provided for all metabolites in every sample. Then, the processed data set was then entered into the SIMCA‐P software package (v13.0, Umetric, Umeå, Sweden). The normalized data were then used to perform analysis.

### Electrophysiological Recordings

Mice were anesthetized with sodium pentobarbital (45 mg kg^−1^) via intraperitoneal injection and subsequently intracardially perfused with an ice‐cold cutting solution composed of (in mm, 209.0 sucrose, 3.1 sodium pyruvate, 22.0 glucose, 1.25 NaH_2_PO_4_, 12.0 sodium l‐ascorbate, 4.9 MgSO_4_‐7H_2_O, and 26.0 NaHCO_3_, which was aerated with 95% O_2_ and 5% CO_2_, pH 7.2–7.4). Coronal slices (300 µm thick) of the DG of the hippocampus were prepared using a vibratome (Leica VT1200 S, Leica Biosystems) and incubated in artificial cerebrospinal fluid consisting of (in mm, 128.0 NaCl, 3.0 KCl, 24.0 NaHCO_3_, 2.0 MgCl_2_, 1.25 NaH_2_PO_4_, 10.0 d‐glucose, and 2.0 CaCl_2_, which was oxygenated with 95% O_2_ and 5% CO_2_, pH 7.2–7.4, 295–305 mOsm) at 28 °C for 1 h. After that, the slices were returned to room temperature for whole‐cell patch‐clamp recordings. Neurons were voltage‐clamped at −70 mV in voltage‐clamp mode to record mEPSCs or mIPSCs. For mEPSC recording, patch electrodes were filled with an internal solution (in mm): 122.5 cesium gluconate, 17.5 CsCl, 0.2 ethylene glycol‐bis(*b*‐aminoethylether)‐*N*,*N*,*N9*,*N9*‐etraacetic acid (EGTA), 10.0 *N*‐(2‐hydroxyethyl)piperazine‐29‐(2‐ethane‐sulfonic acid) (HEPES), 1.0 MgCl_2_, 4.0 magnesium ATP, 0.3 sodium GTP, and 5.0 QX314, pH 7.25 and 280–300 mOsm. Tetrodotoxin (TTX, 10 µm) and bicuculline (20 µm) were included during mEPSC recordings. For mIPSC recordings, pipettes (4–6 MΩ) were filled with an internal solution comprising (in mm) 153.3 CsCl, 1.0 MgCl_2_‐6H_2_O, 5.0 EGTA, 4.0 magnesium ATP, and 10.0 HEPES, adjusted to pH 7.25 with CsOH (280–300 mOsm). mIPSCs were recorded in the presence of TTX (10 µm) and CNQX (10 µm). All data were obtained by pCLAMP 10 (Axon Instruments, Molecular Devices, San Jose, CA) and the MultiClamp 700B amplifier (Molecular Devices, Sunnyvale, CA). Records were low‐pass filtered at 2–20 kHz and digitized at 5–50 kHz (Molecular Devices, Jarvisbio, Wuhan, China).

### Western Blotting

Brain tissue was homogenized using RIPA buffer (Beyotime Biotechnology, Shanghai, China) containing phenylmethylsulfonyl fluoride (1:100) and a proteinase inhibitor cocktail (1:100). The homogenates were then boiled for 10 min, and centrifuged at 14 000 *g* for 10 min. The supernatants were collected, and protein concentrations were quantified using a BCA kit (Thermo Fisher Scientific, Waltham, MA, USA). Proteins from the extracts were separated by 10% sodium dodecyl sulfate–polyacrylamide gel electrophoresis and subsequently transferred to a nitrocellulose membrane. The membranes were blocked with 5% nonfat milk for 1 h and incubated overnight at 4 °C with primary antibodies: anti‐Occludin (#27260‐1‐AP, Proteintech), anti‐Claudin (#MA5‐27605, Thermo Scientific), and anti‐GAPDH (#ab64613, Abcam). Then, the membranes were washed 3 times with TBST for 10 min each and incubated with the secondary antibody at room temperature for 1 h in the dark. Blots were visualized using the Odyssey Infrared Imaging System (Li‐Cor Biosciences, Lincoln, NE, USA), and the protein bands were quantitatively analyzed using ImageJ software (Rawak Software, Germany).

### Immunofluorescence and Fluorescence Imaging

Mice were perfused with a 0.9% saline solution followed by 4% paraformaldehyde in PBS. The brains were postfixed for 24 h in 4% paraformaldehyde and subsequently immersed in a gradient sucrose solution (15–30%) for 3 days at 4 °C. Cryosections (30 µm thick) were incubated in 5% bovine serum albumin and 0.3% Triton X‐100 for 1 h at room temperature, followed by overnight incubation with primary antibodies at 4 °C. Primary antibodies were used as follows: anti‐Iba1 (#019–19741, Wako), anti‐DCX (#91954S, Cell Signaling Technology), and anti‐NeuN (#12943, Cell Signaling Technology). Subsequently, the slices were rinsed and incubated with the appropriate Alexa dye‐tagged secondary antibodies: donkey anti‐mouse 488 (#715‐546‐151, Jackson Immuno Research Labs) and donkey anti‐rabbit 594 (#715‐585‐152, Jackson Immuno Research Labs) for 1 h at room temperature. The slices were then mounted and air‐dried in the dark. All images were observed using the LSM800 confocal microscope (Zeiss, Germany).

### Statistical Analysis

Data were analyzed using GraphPad Prism version 8.0 and presented mean ± standard error of the mean (SEM). To assess the normality of the data, a Kolmogorov–Smirnov test was conducted. For data exhibiting a normal distribution, differences between groups were evaluated by Student's *t*‐test or one‐way analysis of variance (ANOVA). Two‐way repeated‐measures ANOVA followed by Bonferroni's post hoc test, was employed to analyze differences in Sholl analysis. The study was not preregistered; no formal randomization methods were applied, and no blinding was performed. A post‐hoc power analysis was conducted using G*Power to validate the sample size.^[^ ^58^ ^]^ The statistical test method for each figure was indicated in the figure legend. Statistical significance was defined as *p < 0.05, **p < 0.01, ***p < 0.001, and****p < 0.0001. Mice in poor condition (not in health condition and eventually deceased) were excluded from the study.

## Conflict of Interest

The authors declare no conflict of interest.

## Author Contributions

H.Z. and X.Q. contributed equally to this work. H.Z.: writing ‐ original draft, conceptualization, data curation; X.Q.: conceptualization, validation, visualization, formal analysis; H.S.: validation, visualization; J.Z.: investigation, methodology; H.W., L.Z., Y.L., Z.W., Y.Z., Y.L., J.Y., W.H.: validation, visualization, software; Z.C.: supervision; J.Z.: funding acquisition, supervision; Y.J., X.W., R.L.: funding acquisition, conceptualization, supervision, writing ‐ review and editing.

## Supporting information



Supporting Information

## Data Availability

The data that support the findings of this study are available from the corresponding author upon reasonable request.
