# Supplementary material for: A Peripheral Mechanism of Depression: Disturbed Intestinal Epithelial Per2 Gene Expression Causes Depressive Behaviors in Mice with Circadian Rhythm Disruption via Gut Barrier Damage and Microbiota Dysbiosis
Source: Adv Sci (Weinh). 2025 Aug 23;12(43):e01818. doi: 10.1002/advs.202501818 (PMC12631932; doi:10.1002/advs.202501818)
Supplement: Supplementary file 1 — Supporting Information [file ADVS-12-e01818-s001.docx]

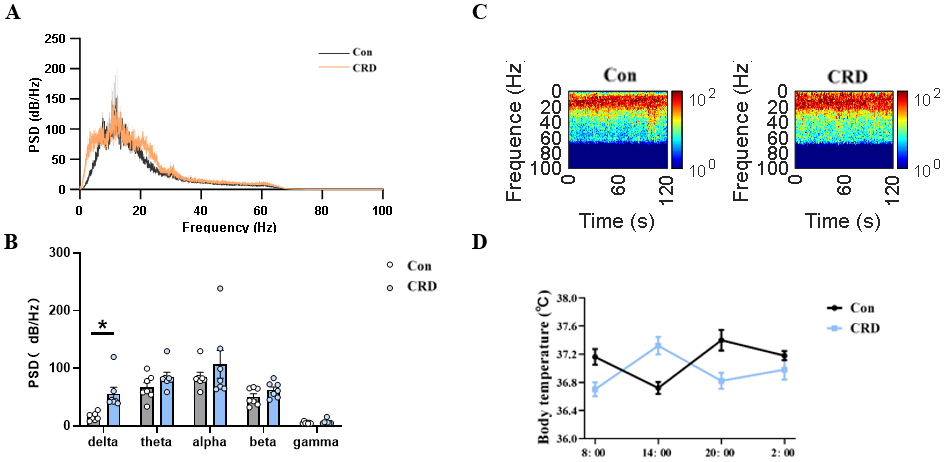


**Supplementary Figure 1 CRD-induced changes of EEG and core body temperature in mice**

(A) Power spectral density (PSD) recording of overall brain cortical discharges in control and CRD mice detected by electroencephalography (EEG) (n=7 for each group); (B) Quantitative analysis of different frequency bands in (A), n=7 for each group; (C) Representative electroencephalogram power spectrograms of the two groups; (D) Rectal temperature was detected using anal thermometer at 8:00. 14:00, 20:00, and 2:00 respectively after 8-week phase delay. n=5 for each group. Data are represented as mean ± SEM. **P* < 0.05.


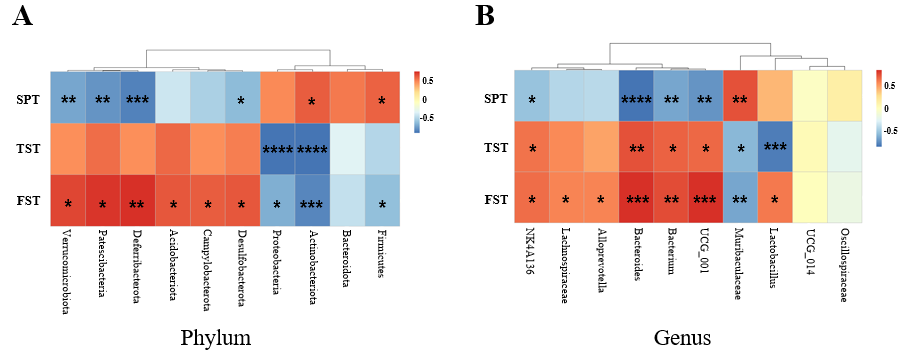


**Supplementary Figure 2. Correlation analysis of differential microbiota and depression-like behaviors**

(A-B) The correlation between depressive-like phenotypes (behaviors in the SPT, TST and FST) and the top 10 differential microbiota (A for phylum, B for genus) was analyzed by Spearman's correlation. The color in the heatmap (blue to red) indicates the different correlation coefficients of Spearman's correlation analysis. **P* < 0.05, ***P* < 0.01, ****P* < 0.001, *****P* < 0.0001.


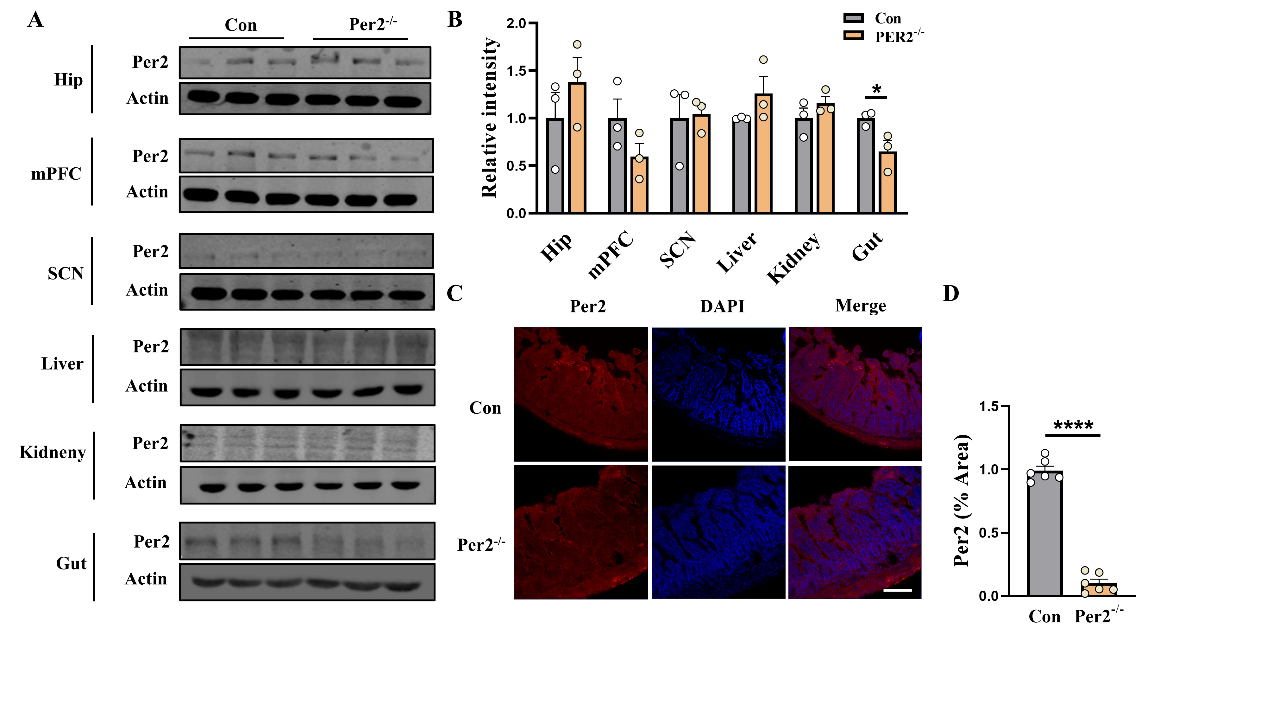


**Supplementary Figure 3. Per2 gene in intestinal epithelium was specifically knocked out in Per2^-/-^ mice**

(A-B) Representative immunoblots and quantitative analysis of Per2 protein levels in the hippocampus, cortex, SCN, liver, kidney, and colon tissue homogenates from Con and Per2^-/-^ mice, n=3 for each group; (C) Representative immunostaining images with antibodies against Per2 (red) to show the Per2 protein in the colonic epithelial cells, counterstained with DAPI (blue). Scale bar: 100 μm; (D) Quantification of the Per2-positive immunostaining area in (C), n=6 for each group; Data are represented as mean ± SEM. **P* < 0.05, *****P* < 0.0001.


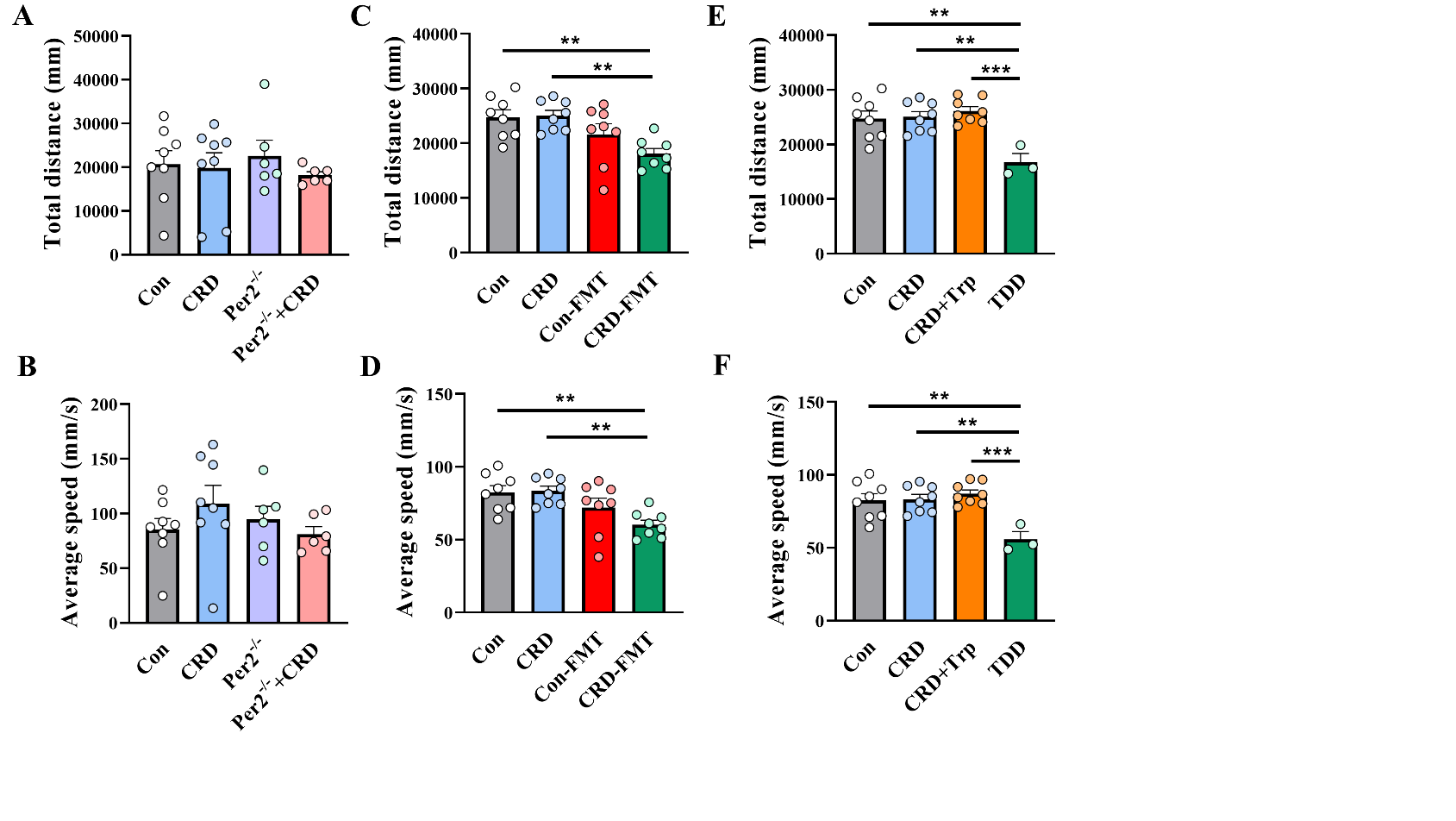


**Supplementary Figure 4 Motor ability of the mice does not differ in different groups**

(A-B) The total distance (mm) and the average speed (mm/s) in OFT in Con, CRD, Per^/-^ and Per^-/-^ + CRD group, n=8 for Con and CRD group, n=6 for Per^-/-^ and Per^-/-^+CRD group; (C-D) The total distance (mm) and the average speed (mm/s) in OFT in Con, CRD, Con-FMT and CRD-FMT group, n=8 for each group; (E-F) The total distance (mm) and the average speed (mm/s) in OFT in Con, CRD, CRD+Trp and TDD group, n=8 for Con, CRD and CRD+Trp group, n=3 for TDD group; OFT, Open- field test. Data are represented as mean ± SEM.***P* < 0.01, ****P* < 0.001.


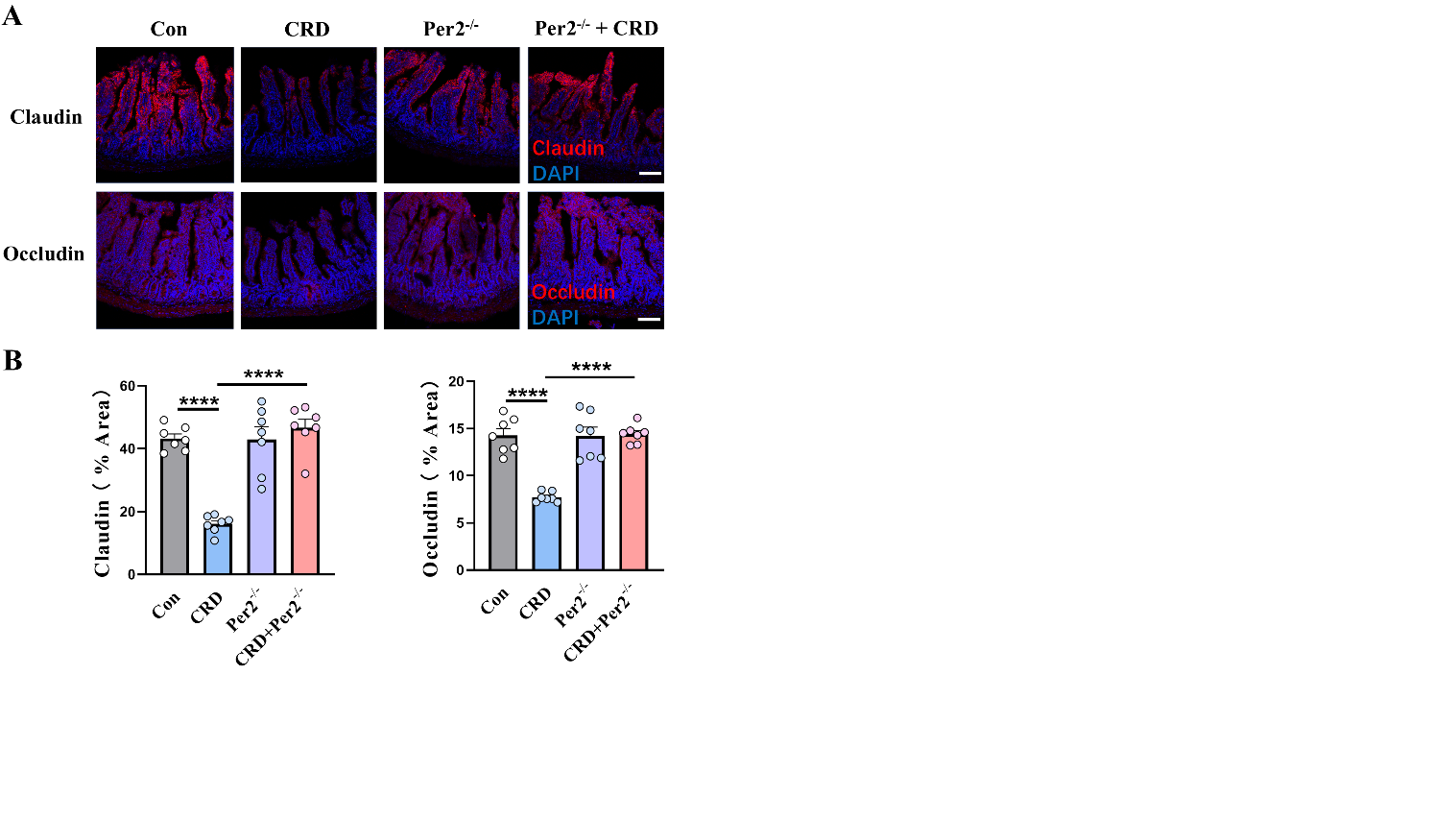


**Supplementary Figure 5 Specific deletion of intestinal epithelial Per2 prevents CRD-induced intestinal barrier damage**

(A) Representative images of Claudin and Occludin immunofluorescence in the intestinal epithelia. Scale bar: 100 μm; (B) Quantification of Claudin and Occludin positive immunostaining area in the four groups of mice, n=7 for each group; Data are represented as mean ± SEM. *****P* < 0.0001.


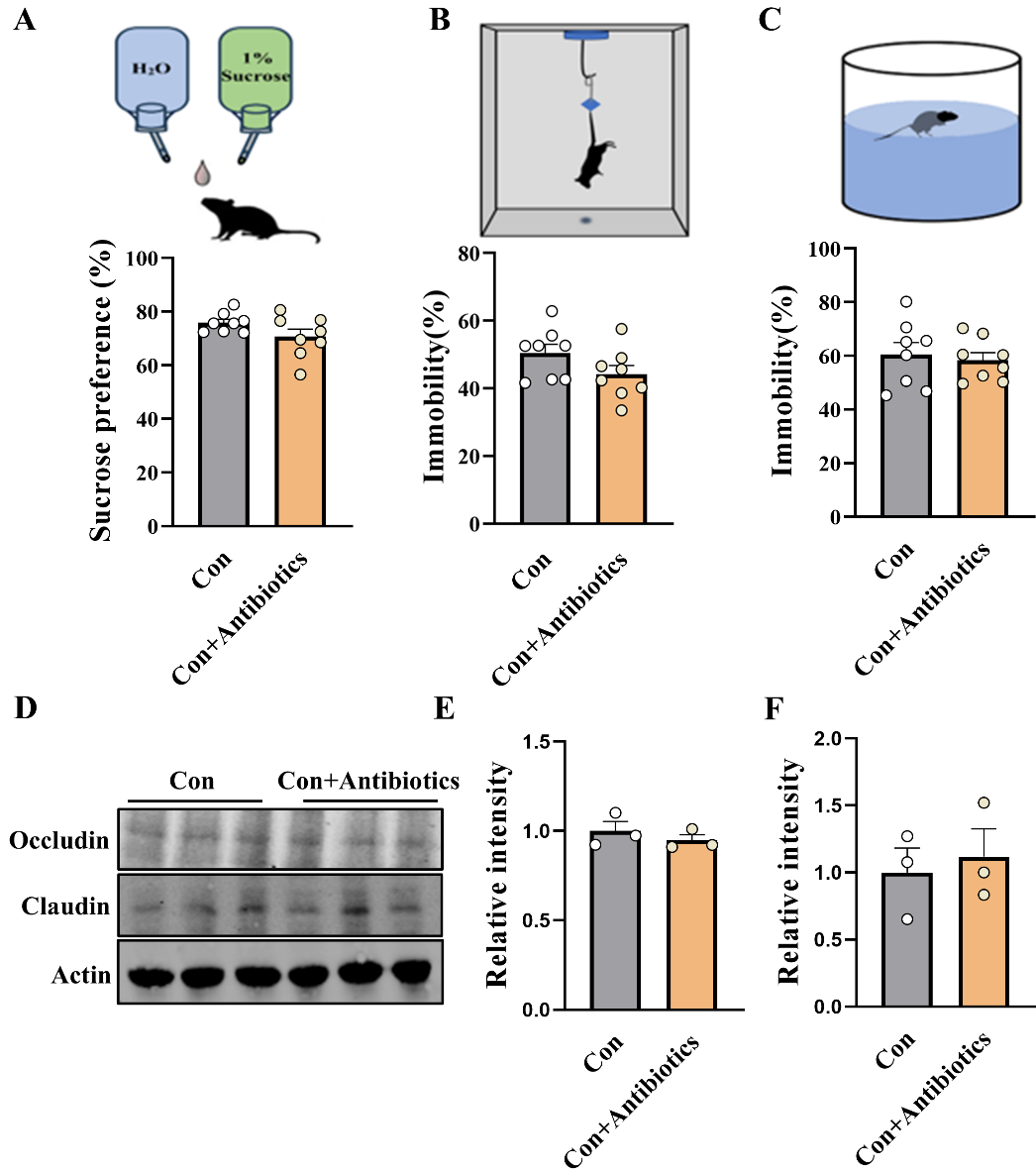


**Supplementary Figure 6. Antibiotics intervention does not induce depressive-like phenotypes and intestinal barrier damage**

(A) The schematic diagram of sucrose preference test (SPT) and the sucrose preference ratio (%) of the mice, n=8 for each group; (B) The schematic diagram of tail suspension test (TST) and the immobility time ratio (%) of the mice, n=8 for each group; (C) The schematic diagram of forced swimming test (FST) and the immobility time ratio (%) of the mice, n=8 for each group; (D-F) Representative immunoblots and quantitative analysis of intestinal barrier protein Occludin and Claudin in colon tissue homogenates, n=3 for each group. Data are represented as mean ± SEM.


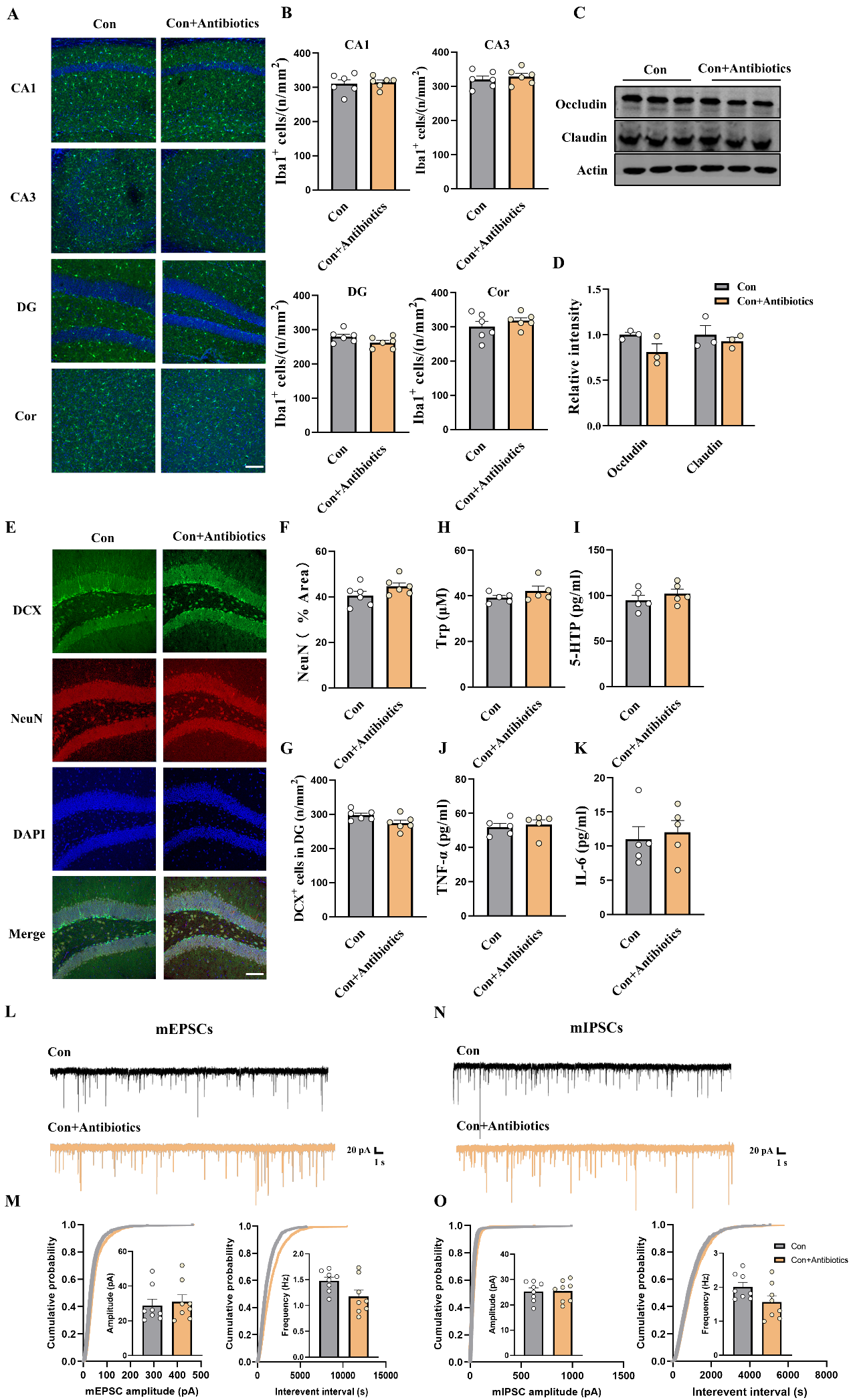


**Supplementary Figure 7. Antibiotics intervention does not induce blood-brain barrier damage, neuroinflammation, and deficits in hippocampal neurogenesis and neurologic function**

(A) Representative images of Iba-1 immunofluorescence in different regions of the hippocampus and cortex. Scale bar: 100 μm; (B) Quantification of Iba-1-positive microglia number in the four groups of mice, n=6 for each group; (C-D) Representative immunoblots and quantitative analysis of barrier protein Occludin and Claudin in hippocampal tissue homogenates, n=3 for each group; (E) Representative immunostaining images with antibodies against DCX (green) and NeuN (red) to show the new-born unmatured neurons and mature neurons in the hippocampal DG, counterstained with DAPI (blue). Scale bar: 100 μm; (F-G) Quantification of the number of DCX^+^ cells and the NeuN-positive immunostaining area in (E), n=6 for each group; (H-I) Quantification of TRP and 5-HTP levels in serum of mice in two groups, n=5 for each group; (J-K) Quantification of TNF- α and IL- 6 levels in serum in two groups, n=5 for each group; (L) Representative mEPSC traces recorded in hippocampal DG; (M) Cumulative distribution of mEPSC amplitude (left) and interevent intervals (right) (n=8 cells from three mice); (N) Representative mIPSC traces recorded in hippocampal DG; (O) Cumulative distribution of mIPSC amplitude (left) and interevent intervals (right) (n=8 cells from three mice). Data are represented as mean ± SEM.


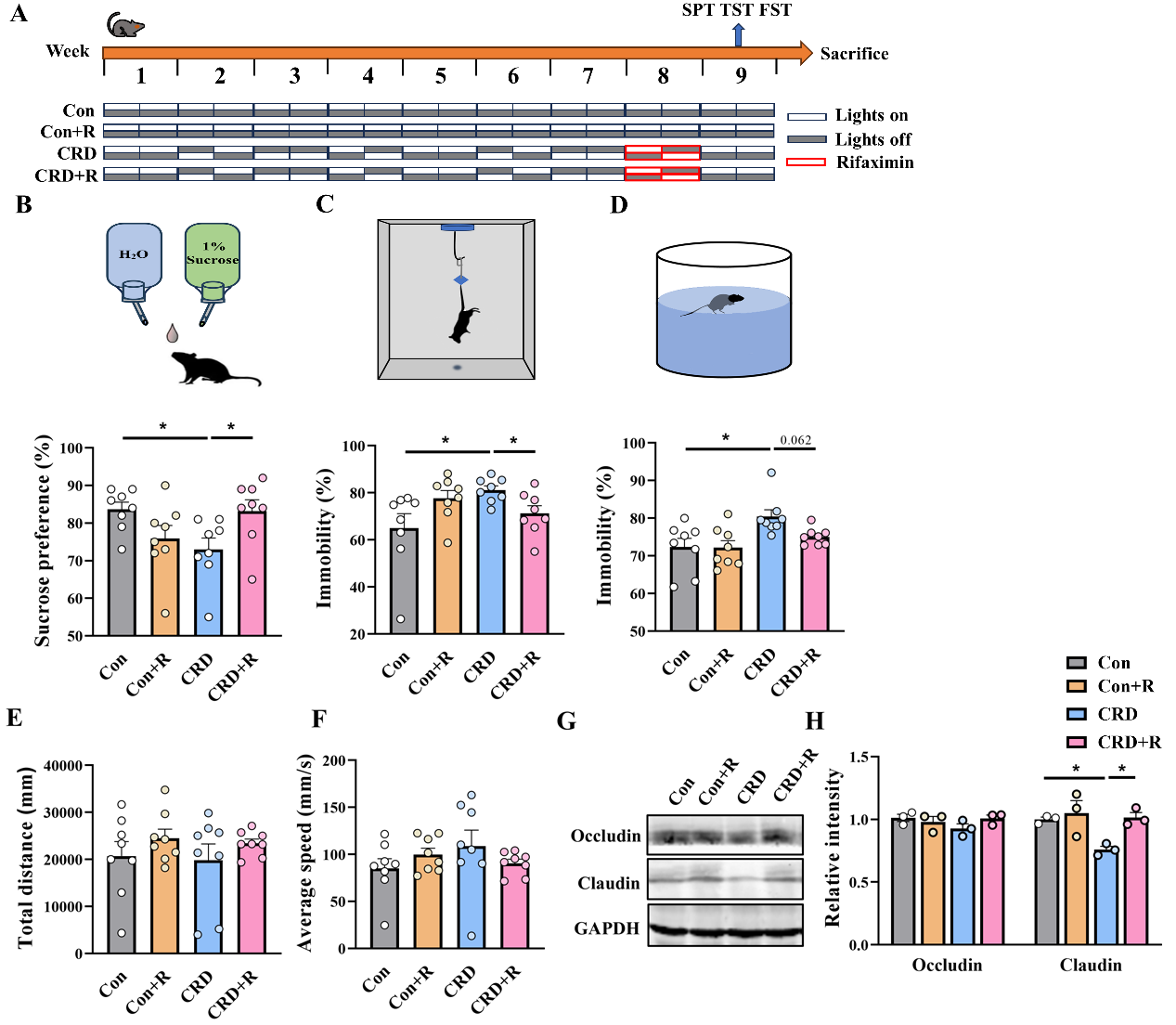


**Supplementary Figure 8 Rifaximin ameliorates CRD-induced depression and impairment of intestinal barrier in mice**

(A) Schematic diagram illustrating the experimental design: control and CRD mice were supplemented with Rifaximin through gavage during the last week of model establishment; (B) The schematic diagram of sucrose preference test (SPT) and the sucrose preference ratio (%) of the mice, n=8 for each group; (C) The schematic diagram of tail suspension test (TST) and the immobility time ratio (%) of the mice, n=8 for each group; (D) The schematic diagram of forced swimming test (FST) and the immobility time ratio (%) of the mice, n=8 for each group; (E-F) The total distance (mm) and the average speed (mm/s) in Open- field test (OFT), n=8 for each group; (G-H) Representative immunoblots and quantitative analysis of intestinal barrier protein Occludin and Claudin in colon tissue homogenates, n=3 for each group. Data are represented as mean ± SEM.**P* < 0.05.


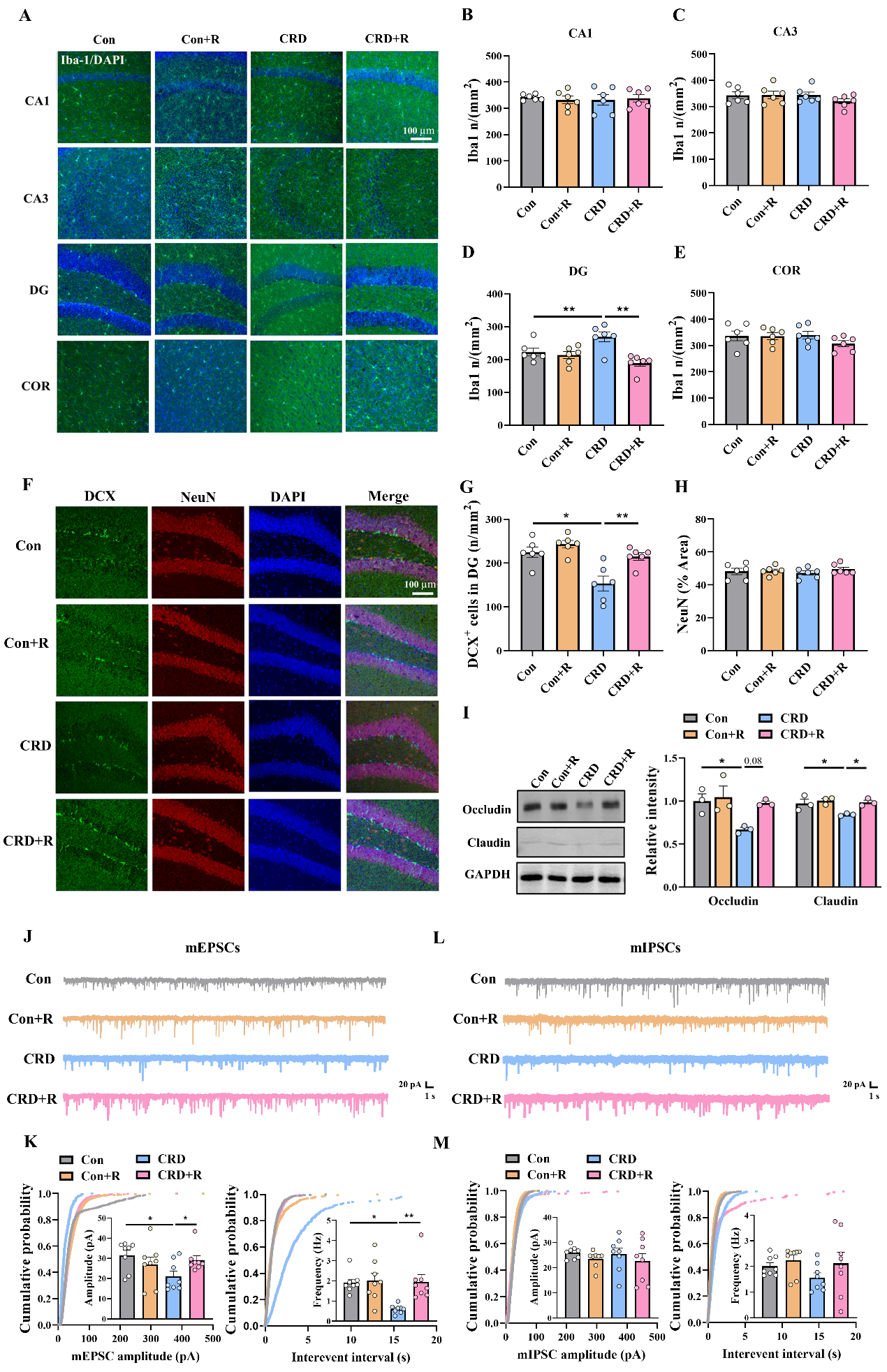


**Supplementary Figure 9 Rifaximin prevents CRD-induced blood-brain barrier damage and neuroinflammation, protects hippocampal neurogenesis and neurological function**

(A) Representative images of Iba-1 immunofluorescence in different regions of the hippocampus and cortex. Scale bar: 100 μm; (B–E) Quantification of Iba-1-positive microglia number in the four groups of mice, n=6 for each group; (F) Representative immunostaining images with antibodies against DCX (green) and NeuN (red) to show the new-born unmatured neurons and mature neurons in the hippocampal DG, counterstained with DAPI (blue). Scale bar: 100 μm; (G-H) Quantification of the area fraction of the number of DCX^+^ cells and the NeuN-positive immunostaining area in (F), n=6 for each group; (I) Representative immunoblots and quantitative analysis of barrier protein Occludin and Claudin in hippocampal tissue homogenates from four groups of mice, n=3 for each group; (J) Representative mEPSC traces recorded in hippocampal DG; (K) Cumulative distribution of mEPSC amplitude (left) and interevent intervals (right) (n=8 cells from three mice); (L) Representative mIPSC traces recorded in hippocampal DG. (M) Cumulative distribution of mIPSC amplitude (left) and interevent intervals (right) (n=8 cells from three mice). CRD, Circadian rhythm disruption; mEPSCs: miniature excitatory postsynaptic currents; mIPSCs: miniature inhibitory postsynaptic currents; Data are represented as mean ± SEM. **P* < 0.05, ***P* < 0.01.


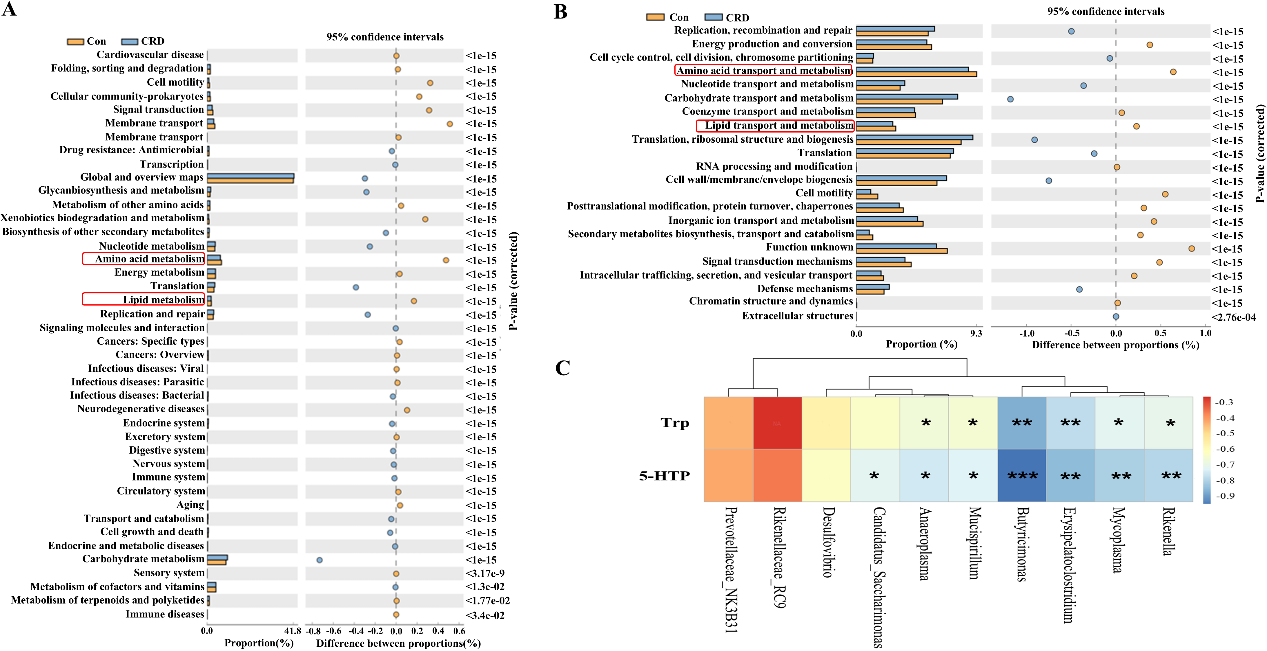


**Supplementary Figure 10. CRD - related differential gut microbiota is strongly associated with tryptophan metabolism**

**(A**) Observation of differences and variations in metabolic pathways of functional genes in differently grouped microbial communities, the proportion of abundance of different functions in the two samples is shown on the left in the image, the proportion of differences in functional abundance within the 95% confidence interval is shown in the middle, and the rightmost value is the *P*-value; **(B)** The Clusters of Orthologous Groups of proteins (COG) functional prediction analysis method responds to the distribution and occupied abundance of functions of the sequences in the samples, with the proportion of abundance of different functions in the two samples shown on the left, the proportion of differences in functional abundance within the 95% confidence intervals shown in the middle, and the value on the far right being the *P*-value; **(C)** The correlation between metabolites (Trp and 5-HTP) and the differential microbiota genus was analyzed by Spearman's correlation. The color in the heatmap (blue to red) indicates the different correlation coefficients of Spearman's correlation; **P* < 0.05, ***P* < 0.01, ****P* < 0.001.


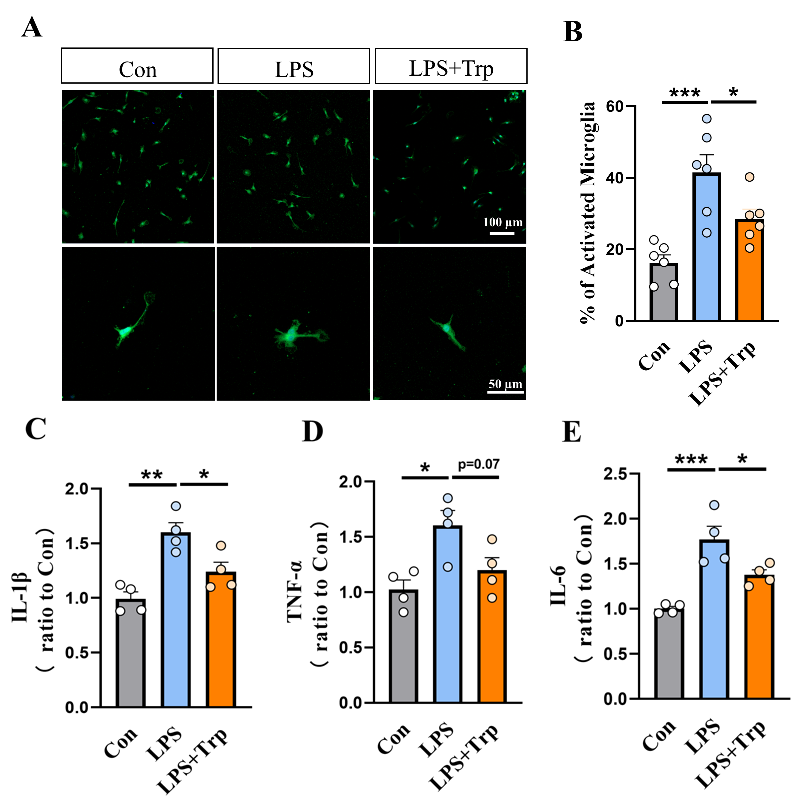


**Supplementary Figure 11. Tryptophan alleviates LPS-induced inflammatory response in primary microglial cells**

(A-B) Representative immunostaining images with antibodies against Iba-1 (green) to show the primary microglia and quantification of the proportion of activated microglial cell, Scale bar: 100 μm (Upper) and 50 μm (lower), n=6 cells per group; (C-E) IL-1β, TNF- α and IL-6 levels, n=4. Data are represented as mean ± SEM. **P* < 0.05, ***P* < 0.01, ****P* < 0.001.
